# Molecular Mechanisms of Migraine: Nitric Oxide Synthase and Neuropeptides

**DOI:** 10.3390/ijms241511993

**Published:** 2023-07-26

**Authors:** Nazia Karsan, Helin Gosalia, Peter J. Goadsby

**Affiliations:** 1 Headache Group, NIHR King’s Clinical Research Facility and SLaM Biomedical Research Centre, The Wolfson Sensory, Pain and Regeneration Research Centre, Institute of Psychiatry, Psychology and Neuroscience, King’s College London, London SE5 9PJ, UK; nazia.karsan@kcl.ac.uk (N.K.); helin.1.gosalia@kcl.ac.uk (H.G.); 2 Department of Neurology, University of California, Los Angeles, CA 90095, USA

**Keywords:** migraine, nitric oxide, neuropeptide, calcitonin gene related peptide, pituitary adenylate cyclase activating polypeptide, Neuropeptide Y, vasoactive intestinal peptide

## Abstract

Migraine is a common condition with disabling attacks that burdens people in the prime of their working lives. Despite years of research into migraine pathophysiology and therapeutics, much remains to be learned about the mechanisms at play in this complex neurovascular condition. Additionally, there remains a relative paucity of specific and targeted therapies available. Many sufferers remain underserved by currently available broad action preventive strategies, which are also complicated by poor tolerance and adverse effects. The development of preclinical migraine models in the laboratory, and the advances in human experimental migraine provocation, have led to the identification of key molecules likely involved in the molecular circuity of migraine, and have provided novel therapeutic targets. Importantly, the identification that vasoconstriction is neither necessary nor required for headache abortion has changed the landscape of migraine treatment and has broadened the therapy targets for patients with vascular risk factors or vascular disease. These targets include nitric oxide synthase (NOS) and several neuropeptides that are involved in migraine. The ability of NO donors and infusion of some of these peptides into humans to trigger typical migraine-like attacks has supported the development of targeted therapies against these molecules. Some of these, such as those targeting calcitonin gene-related peptide (CGRP), have already reached clinical practice and are displaying a positive outcome in migraineurs for the better by offering targeted efficacy without significant adverse effects. Others, such as those targeting pituitary adenylate cyclase activating polypeptide (PACAP), are showing promise and are likely to enter phase 3 clinical trials in the near future. Understanding these nitrergic and peptidergic mechanisms in migraine and their interactions is likely to lead to further therapeutic strategies for migraine in the future.

## 1. Introduction

Migraine is a complex brain disorder characterised by recurrent attacks of headache as well as other symptoms, such as sensory sensitivities, nausea and vomiting, cognitive dysfunction, and altered arousal [1]. A proportion of those with migraine will experience aura symptoms associated with attacks, which are typically visual and can include sensory and speech symptoms, as well as brainstem and hemiplegic symptoms [2]. Whilst a family history of migraine is often present in those afflicted (estimated to be around 42% [3]), genome-wide association studies (GWAS) of migraine have revealed a total of more than 180 single-nucleotide polymorphisms (SNP’s) or mutations within genes that may be implicated in migraine [4,5,6,7,8,9], with a suggestion that heritability is higher in migraine with than without aura [10]. Monogenic forms of migraine, such as familial hemiplegic migraine (FHM), are considerably rarer than the more common polygenic form and tend to cause more severe aura phenotypes, as well as other associated symptoms [11]. A significant number of the mutations implicated in these disorders cause a change in ion channel function or alter synaptic neurotransmission, thereby increasing cortical hyperexcitability. Neuronal and smooth muscle cell types have been implicated through genes encoding these functions, and these may mediate a threshold to cortical spreading depression (CSD), the presumed neurophysiological correlate to migraine aura, as well as affect vascular function [12].

## 2. Basic Pathophysiology

Migraine is well recognised as being a neurovascular disorder, in which primarily neural dysfunction in areas such as the brainstem, hypothalamus, and basal ganglia structures [13], with subsequent secondary vascular involvement, causes the heterogeneous neurological phenotype [14]. The links between aura and headache biologically remain debated, as aura is thought to be a cortical phenomenon that typically occurs before or with headache [15], but can occur at any time in the migraine cycle [2,16], be prolonged [17] or persistent [18], and affects only 30% of those with migraine [19]. Indeed, it is more likely even for those with migraine with aura to experience headache attacks without aura associated [10]. Genetics have therefore contributed to the understanding of migraine mechanisms and possible drug targets, particularly in migraine with aura, but more common migraine is polygenic in nature. Much remains to be learned about how and if the same mechanisms are involved in migraine without aura, and what the associations between aura and headache are.

Additional understanding of the mechanisms of disease in migraine has come from an appreciation of the molecular biology of migraine. This has been recently contributed to by the increase in the development and use of experimental provocation models of human migraine [20]. The ability to use an exogenous compound to trigger phenotypically similar migraine-like attacks to spontaneous ones, to observe these and investigate treatment effects, as well as to conduct repeated measures imaging protocols during these attacks, have been important advances to the migraine research space. These human models have provided an opportunity to further develop an understanding of migraine molecular chemistry and, therefore, the development of drug targets.

## 3. Migraine Triggering Models

Whilst some models have been used for some time, such as the nitroglycerin (NTG) model, which triggers migraine through nitric oxide (NO) mechanisms [21], others have followed more recently, and have already led to important improvements in migraine therapeutics. One such provocation agent is calcitonin gene-related peptide (CGRP), a neuropeptide widely expressed in brain regions of interest in migraine as well as in the cerebral vasculature, and which has had a demonstrated role in migraine since the 1980s [22,23,24]. Its proposed roles in migraine biology and subsequently identified ability to provoke migraine-like attacks when infused into humans [25] contributed to the development of targeted treatments against this pathway, which are now in clinical use in many countries for the acute and preventive management of migraine [26]. Similarly, pituitary adenylate cyclase activating polypeptide (PACAP), another neuropeptide in the vasoactive intestinal peptide (VIP) family, demonstrated to have a role in migraine and cluster headache possibly via the parasympathetic cranial outflow pathway in the sphenopalatine ganglion (SPG) and superior salivatory nucleus (SSN) [27,28,29,30], has also been shown to provoke migraine-like attacks when infused into patients with migraine [31]. Similarly to CGRP, targeted treatments against this pathway have shown preclinical [32] and clinical [33] promise in migraine treatment. Whilst VIP has historically not been thought to provoke migraine-like attacks despite causing vasodilatation [34], there is now current evidence to the contrary [35]. Interestingly, these triggering compounds all cause extracranial dilatation and delayed headache in patients with migraine [36,37], though using similar imaging methodologies, these vascular changes are not present in spontaneous attacks [38]. Aura is infrequently triggered with them, even in those with migraine with aura [31,35,39,40] or familial hemiplegic migraine [41,42,43,44].

Additional triggering compounds of interest have emerged more recently within the calcitonin-CGRP family and include amylin [45] and adrenomedullin [46]. Levcromakalim, an ATP-sensitive potassium channel opener, can also trigger migraine-like attacks and, interestingly, also aura [47,48], and this has alluded to additional downstream neurochemical pathways implicated in migraine pathophysiology and potential therapeutic targets. Despite considerable research in migraine therapeutics, there remains a paucity of specific and targeted treatments available, and many patients remain underserved by current options. The increased understanding of disease mechanisms, therapeutic substrates, and therapeutic development is therefore vital to improving the lives of those afflicted.

## 4. Functional Neuroimaging

Functional neuroimaging work in migraine has identified headache-related activation in brain areas containing nuclei, such as the locus coeruleus (LC), dorsal raphe nuclei (DRN), and hypothalamus [49,50,51,52]. These areas are rich in aminergic fibres and project to other areas involved in trigeminovascular processing in migraine, such as the trigeminal nucleus caudalis (TNC), which express CGRP and PACAP [14]. Modulation of trigeminovascular processing may also involve other receptors, such as GABA, glutamate, cannabinoid, and opioid receptors [14]. For example, the expression of GABA_A_ subunit mRNAs has previously been detected with an overlapping distribution within LC and DRN [53,54], and may therefore regulate serotoninergic and noradrenergic neuronal activity in these regions in migraine. Broad dysfunction within aminergic, peptidergic, and other neurotransmitter networks is likely to be involved in the heterogeneous phenotype.

This review will select NO and some neuropeptides of interest and review their speculated role in migraine, the evidence for this, and the potential for future therapeutics. This will be preceded by a brief introduction to the current understanding of migraine biology and anatomy. This review cannot comprehensively cover all the neuropeptides implicated in migraine biology, so for ease of reading and conciseness, a selection has been chosen.

## 5. Migraine Pathophysiology

The quality and features of migraine headaches are thought to be caused by meningeal nociceptor activation, whose axons originate in the trigeminal ganglion and innervate dural vasculature [55]. Mechanical or electrical dural stimulation has been shown to cause vasodilatation and increased cerebral blood flow and axonal terminal neurochemical release of neuropeptides, such as CGRP, substance P, neurokinin A, VIP, and PACAP [56,57,58]. Nociceptive information is relayed from craniovascular structures via the trigeminal nucleus caudalis (TNC) and its cervical extension (the TCC; trigeminocervical complex), and from here via ascending projections through the brainstem, hypothalamic and thalamic areas, and ultimately to the cerebral cortex for sensory processing. These areas include the noradrenergic locus coeruleus (LC) in the pons, the serotoninergic dorsal raphe nucleus (DRN) in the periaqueductal grey (PAG), the raphe magnus (NRM) in the medulla, and the dopaminergic ventral tegmental area (VTA) and A11 nucleus within the hypothalamus. There are also descending modulatory pathways via brainstem nuclei involved, as well as a trigeminal autonomic reflex mediated by a parasympathetic reflex loop via the sphenopalatine ganglion (SPG) and superior salivatory nucleus (SSN) in the pons to dural vasculature [59]. This pathway mediates the cranial autonomic symptoms (CASs), including lacrimation, conjunctival injection, rhinorrhoea, and aural discomfort, that patients with primary headache disorders can report. A projection from the TCC to the SSN is thought to link the sensory nociceptive and autonomic pathways in migraine [59].

The release of neuropeptides from trigeminal sensory fibres is thought to contribute to vasodilatation, as well as cause other perivascular changes and a cascade of events that ultimately activate and sensitise the trigeminovascular system and promote headache maintenance [14]. Activation of the trigeminovascular system in experimental settings causes the release of CGRP, substance P, neurokinin A, PACAP, and NO from trigeminal sensory nerve fibres, as well as VIP, PACAP and NO, and other neurotransmitter releases from parasympathetic fibres innervating the cranial vasculature [60]. Migraine is, therefore, a neurovascular disorder, and the interaction between the innervation of the cranial vasculature and the effect of subsequent neuropeptide and neurotransmitter release is vital to the perception of head pain, the CAS, which can be associated with migraine, as well as other associated symptoms. It is clear that brainstem and diencephalic regions are activated on brain imaging in migraine, both during headache [49,50,51] and before headache onset during premonitory symptoms [13,61,62,63,64], which in some patients can warn of impending headache [65]. These brain areas correlate with aminergic symptoms such as altered arousal and cognition, yawning, nausea, and food cravings that patients commonly report during this time [1]. The involvement of vasoactive neuropeptides and aminergic brainstem nuclei provides a backdrop for the molecular neurochemistry of this complex disorder.

We will now discuss NO and some of the neuropeptides implicated in migraine and their therapeutic potential for migraine treatment.

## 6. Nitric Oxide (NO)

### 6.1. Preclinical Evidence for NO Mechanisms in Migraine

Nitric oxide (NO) has many neurophysiological functions and is a potent vasodilator. The role of NO has been supported by preclinical models of migraine [66]. A single subcutaneous injection of NTG into rats causes neuronal activation in several brain regions known to be implicated in migraine biology, such as the periaqueductal grey (PAG) and TNC [67], and also sensitises central trigeminocervical neurons [68] and peripheral trigeminal afferents [69]. There is activation of brainstem and hypothalamic areas in rodents prior to TNC activation following NTG exposure [67,70], and central trigeminovascular excitation is at the level of the thalamus and the TCC [71]. Infusion of NTG into the TNC or onto the pial surface promotes the release of CGRP, substance P, and neuronal nitric oxide synthase (nNOS) and subsequent vasodilatation [71,72], suggesting that activation of the NO system promotes vasoactive neuropeptide release from perivascular nerve terminals. NOS is the enzyme, of whichall isoforms promote NO production.

Central neuropeptide responses can be inhibited by sumatriptan [68], olcegepant, a small molecule CGRP antagonist, and a 5HT_1F_ antagonist (ditan) [73], all drugs proven to be clinically efficacious in migraine [74]. L-NGmethylarginine hydrochloride (546C88) (L-NAME), a non-specific NOS inhibitor, sumatriptan, and indomethacin, when administered as pre-treatment, can all inhibit TNC activation caused by NTG administration or electrical stimulation of the superior sagittal sinus (both experimental animal models of migraine) [67,71,75]. Many migraine drugs may therefore exert a therapeutic action via NO- and CGRP-mediated pathways. In animal models, NTG also mediates facial and hind-paw hypersensitivity to cutaneous stimulation, and this neuronal sensitisation is the likely correlate for migraine-related allodynia [68], and it can provoke other migraine-related symptoms, such as anxiety and altered social behaviour [76]. Repeated exposure to NTG has also been used pre-clinically as a model of chronic migraine and sensitisation, and increased periorbital and hind paw hypersensitivity has been demonstrated [77]. An electrophysiology study using an NO donor model (sodium nitroprusside) showed that there is an initial increase in basal firing of meningeal nociceptive neurons following drug exposure and, thereafter, a delayed and prolonged facilitation [78]. Olcegepant reverses this effect at the level of second-order trigeminovascular neurons [79]. There is further support for NTG causing delayed facilitation of basal trigeminal tone and for sensitisation of sensory responses to cutaneous stimulation during this time [68,73]. This is analogous to the initial headache observed in humans during NTG infusion and the delayed migraine-like headache following some hours later [80].

The interaction between NO and CGRP mechanisms is thought to facilitate the role of NO in migraine. There is evidence that NO can directly increase CGRP release via the TRPV1 receptor [81], although clinical trial evidence does not support a role for TRPV1 blockade in migraine [82], and CGRP may cause vasodilatation via NO mechanisms [83]. nNOS promotes CGRP release from trigeminal fibres, and CGRP activates NO production via endothelial NOS (eNOS) [84]. This bidirectional interaction between NO and CGRP mechanisms is likely, therefore, to be important in migraine.

### 6.2. Human Evidence for NO Mechanisms in Migraine

The NTG experimental model has been the most widely used in human migraine research. The realisation that NTG can cause headaches, particularly amongst those with underlying migraine, most likely via NO mechanisms [85,86,87], led to the development of triggering models using NTG in migraine experimental research. NTG can provoke mild headaches that are throbbing and movement sensitive, but lack other migraine features amongst healthy controls [21]. In patients with migraine, NTG infusion leads to a more severe and persistent headache that fulfils diagnostic criteria for migraine without aura, and the migraine-like headache typically occurs at a delay of some hours following infusion [88,89]. It is important to note that the majority of, but not all, patients with migraine will trigger attacks of migraine without aura with NTG [80], suggesting that NO is unlikely to have a key role in aura.

NTG is a potent vasodilator that is thought to be converted into NO in the endothelial layer of vascular walls as its mechanism for the triggering of headaches [90]. NTG-triggered migraine is not associated with vascular dilatation in the cerebral or meningeal circulation [91]. The migraine-like attack that follows NTG exposure can be effectively treated with sumatriptan [92], which is a 5HT_1B/1D_ receptor agonist and causes vasoconstriction, but has primarily neural actions in migraine abortion [93]. L-NGmethylarginine hydrochloride (546C88) effectively aborts acute migraine attacks [94]. Unfortunately, in clinical studies of iNOS inhibitors in migraine, there has been no evidence of an acute [95] nor a preventive [96] effect. There is a similar suggestion of the failed effect of a mixed nNOS inhibitor/5HT_1B/1D_ receptor agonist when taken during the aura phase, although this study was limited by a high drop-out rate and underpowering due to the small remaining sample size [97].

Raised nitrite [98] and CGRP [23,99] levels have been demonstrated in the central circulation during a migraine attack, and CGRP levels normalise following sumatriptan administration [100], akin to during spontaneous migraine attacks [23,24]. Importantly, as well as triggering headaches, NTG infusion can also provoke premonitory symptoms, which occur before headache and in the absence of headache and allude to widespread brain dysfunction [13,61,62,80,101]. There must therefore be a downstream mechanism of NTG and NO in migraine beyond the cerebral vasculature, given that intravenous NTG has a half-life of 3–4 min [102], yet these symptoms and associated brain changes occur later.

### 6.3. Therapeutic Scope

There is understandable interest in NOS inhibition in migraine therapeutics. All the enzyme isoforms in the NOS family increase the production of NO. Alongside the suggestion of non-specific NOS inhibition in migraine therapeutics [94], preclinical studies suggest that targeting specific enzymes in the family may also hold promise without the unwanted side effects of targeting all the enzymes.

A study demonstrated a combined nNOS inhibitor and 5HT_1B/1D_ agonist could inhibit CGRP release from trigeminal ganglion and TNC [103], although it was not effective in a clinical trial [104]. NTG also upregulates dural mRNA for iNOS, as well as plasma protein leakage, both inhibited by iNOS inhibition [105]. Unfortunately, a specific iNOS inhibitor failed in both acute [95] and preventive [96] clinical trials in migraine.

Further understanding of the specific NOS isoforms and their roles, as well as targeted inhibition, may hold relevance in future migraine therapeutics.

## 7. Calcitonin Gene-Related Peptide (CGRP)

### 7.1. Preclinical Evidence for CGRP in Migraine

CGRP has been a subject of interest in migraine for some time, following initial studies showing its release into the central circulation in an animal model of migraine [22,106]. The CGRP receptor has three protein components: the receptor activity modifying protein 1 (RAMP1), the calcitonin receptor-like receptor (CLR), and the receptor component protein (RCP) [107]. Differing dimerisations produce different receptor types with varying affinities for CGRP.

The anatomy of CGRP (largely α-CGRP) expression has been mapped in animals and is shown to involve several areas of interest in migraine pathophysiology, such as the brainstem, TCC, trigeminal ganglion [108,109,110], as well as SPG [111]. NOS and other neuropeptides are also expressed here, and the SPG may be a site for the interaction between sensory and parasympathetic pathways in migraine [112]. There are CGRP projections from sensory trigeminal ganglion to the cerebral and dural vessels (via C and Aδ fibres) and to the spinal cord, but not to meningeal trigeminal afferents [109,110]. In the trigeminal ganglion, CGRP is co-expressed with serotonin, PACAP, and NOS [113,114]. The satellite glial cells of the trigeminal ganglion express RAMP1 and CLR [110], and the neuron-glial interaction may be important in migraine and peripheral sensitisation and be mediated via CGRP in part [115]. Satellite glial cells are activated by CGRP release and cause further release of proinflammatory cytokines, augmenting the neuronal response [115]. Indeed, it has been shown that one site of action for CGRP in the trigeminovascular system may be the nodes of Ranvier [116].

In animal models, stimulation of the trigeminal ganglion, which innervates the cranial vasculature, causes the release of substance P and CGRP from the perivascular nerve terminals [24], causing vascular dilatation that is inhibited by the triptans [117]. Whilst CGRP is a vasodilator like NTG, it is a large peptide in molecular structure and, therefore, may not penetrate the blood–brain barrier well in the same way as NO, and may exert at least some of its actions in migraine peripherally. CGRP vasodilatation can be NO-mediated, as discussed previously, via phosphokinase A-mediated activation of eNOS, but it can also occur independently of NO mechanisms via direct CGRP binding onto vascular smooth muscle [118].

In addition, CGRP has neuromodulatory roles, including effects on the glutaminergic system [119]. Locally applied CGRP causes trigeminovascular [119] and thalamic [120] activation of neurons with nociceptive inputs, which can be inhibited by CGRP receptor antagonists olcegepant or CGRP_8-37_, respectively. This is a potential mechanism for central sensitisation in migraine that may be mediated by CGRP. Similar responses have been demonstrated when CGRP is locally applied to PAG in rodent models, where CGRP causes activation of dural nociceptive trigeminovascular neurons, and this effect can be reversed by olcegepant applied in the same area [121].

A CGRP-sensitised mouse has also been developed as a preclinical migraine model [122,123,124] and demonstrates the role of CGRP in migraine-related behaviours apart from headaches, like photophobia. This effect may occur via third-order neurons in the thalamus [120,125]. CGRP may exert its action in migraine via a range of mechanisms, such as peripheral sensitisation, both through vasodilatation and additional potential indirect effects of CGRP on plasma extravasation, as CGRP stimulates substance P release, which, along with neurokinin A, are the main mediators of plasma extravasation that cause activation of meningeal nociceptors [126]. Importantly, plasma extravasation blockers [127] and substance P/neurokinin-1 receptor antagonists [128] have proven ineffective in clinical trials for migraine. Mast cell degranulation, initiation of a cyclic AMP signalling cascade, central sensitisation via glutamatergic signalling, and CSD may be further mechanisms of CGRP actions in migraine [129].

### 7.2. Clinical Evidence for CGRP in Migraine

As discussed above, alongside the release of NO during acute migraine attacks, other vasoactive neuropeptides, such as CGRP, have been historically shown to be increased in the central circulation during acute migraine attacks in humans [23]. Further studies have demonstrated elevated saliva and blood CGRP levels during both spontaneous and triggered attacks [130,131] and a reduction in CGRP levels following treatment correlating with headache intensity. Interictal serum CGRP levels have been demonstrated to be elevated in both episodic [132] and chronic migraine [133]. In those with migraine, as is seen with NTG, CGRP can provoke migraine without aura attacks [25,40]. Healthy volunteers experience non-migrainous head discomfort or fullness following CGRP exposure [40,134]. There is a suggestion that novel treatments targeting the CGRP pathway may be more efficacious in those who successfully trigger migraine-like headaches with CGRP infusion [135]. This concept may prove to be the basis of potentially using provocation models for treatment response prediction. Akin to NTG, not all patients with migraine trigger with CGRP, and interestingly, the triggering of premonitory symptoms is reported to be less common than with NTG [136], although this may have a clinical explanation, and aura is not commonly triggered [42,44]. CGRP-triggered migraine-like attacks respond to sumatriptan treatment [137]. Another possible link to migraine relates to the circadian variation in CGRP levels [138,139]. The late morning changes correlate with the relative distribution of migraine attacks throughout the day [140], which has a distinct predilection in large data sets for late morning.

### 7.3. Therapeutic Scope

Recently, CGRP-targeted therapies have reached clinical practice and are changing the landscape of migraine treatment for the first time since the triptan era, by providing specific and targeted acute and preventive treatments for migraine. The small molecule CGRP antagonists, the *gepants*, of which seven have been synthesized, have all shown clinical efficacy. Initial concerns of liver toxicity led to the halting of telcagepant and MK-3207 development [141,142,143,144,145]. Olcegepant was the first one formulated, but it was in intravenous form and was never commercialised [146]. Subsequently, rimegepant [147,148,149,150], ubrogepant [151,152,153,154,155,156], atogepant [157,158,159,160], and zavegepant [161,162] have demonstrated clinical efficacy without liver toxicity [163]. Rimegepant offers an opportunity to utilise the quantum between acute and preventive therapy in migraine, with the same drug being effective acutely and preventively. Importantly, post hoc analyses of the *gepant* trials show the drugs are well tolerated in those with vascular disease [148,151] and are effective in participants who report having not responded to triptans [164,165]. Interestingly, there is emerging evidence that ubrogepant may be useful in preventing headache onset when taken during the premonitory phase and also in reducing non-headache-related attack burden [166,167].

Preventively, monoclonal antibodies targeting the CGRP peptide (galcanezumab, eptinezumab, and fremanezumab) or canonical receptor (erenumab) have been developed for migraine treatment [168,169,170,171,172,173,174,175,176,177,178], and again all have shown unanimous efficacy without significant side effects or safety concerns. Galcanezumab has been demonstrated to be able to reduce the burden from premonitory symptoms, triggers, and aura following 3 months of use [179]. The site of action of these agents and of the *gepants* remains unclear [180]. Whilst an intact blood–brain barrier in migraine has been demonstrated in imaging studies macroscopically [181,182,183], and it has been suggested that CGRP-targeted therapies exert their action peripherally [184], there is emerging evidence for central functional imaging effects of the large molecular size monoclonal antibodies [185] and for clinical effects of these drugs on symptoms that could only be deemed as centrally neurally driven, such as cognition and fatigue [186], and premonitory symptoms, triggers and aura [179]. Moreover, it has been known for some decades that immunoglobulins can be found in the cerebrospinal fluid [187], so it remains to be seen if CGRP monoclonal antibodies can be detected centrally; we think this is highly likely.

It seems that CGRP probably has important roles in migraine both peripherally, via its release from sensory neurons innervating blood vessels, causing vasodilatation and peripheral sensitisation. Similarly, a role through central mechanisms via expression in several brain areas important in migraine, therefore contributing to central sensitisation, sensory aversion, and CSD [188], may be of equal, or perhaps, greater, importance.

CGRP-targeted therapies provide an exciting new option in migraine therapeutics. It is vital to remember that not all patients with migraine will respond to these therapies, and there are, therefore, likely to be other mechanisms, environmental, genetic, and epigenetic factors [189] at play mediating the threshold to migraine. Further work into other CGRP-related receptor subtypes, aside from the canonical CGRP receptor, such as the amylin and adrenomedullin receptors, is warranted, given an amylin analogue and adrenomedullin can both trigger migraine-like attacks in those with migraine [45,46].

Despite the significant interest in CGRP in migraine over recent times sparked by the introduction of exciting new targeted therapies in clinical practice, recent years have also produced the identification of and interest in several other migraine mechanisms apart from CGRP. Some of these involve other neuropeptides, and some involve other non-NO intracellular targets and ion channels [190]. Some of the non-CGRP peptidergic mechanisms will be discussed here.

## 8. Pituitary Adenylate Cyclase Activating Polypeptide (PACAP)

### 8.1. Preclinical Evidence for PACAP in Migraine

PACAP is expressed throughout the central nervous system and peripheral tissues, including those involved in migraine biology [56,191,192], and in particular by parasympathetic fibres extracranially and in the SPG [112]. PACAP binds to three different G-protein coupled receptors: the VPAC1, VPAC2, and PAC1 receptors [193]. The first two also bind vasoactive intestinal peptide (VIP), another potent vasodilatory neuropeptide in the same family. The PAC1 receptor is more specific to PACAP and has therefore had the most interest in migraine, particularly given initial suggestions that VIP was not implicated in the pain part of migraine [34]. PACAP and VIP, as well as being from the same neuropeptide family and acting on similar receptors, also functionally interact; PACAP38 fibres innervate VIP neurons [194], and PACAP38 promotes VIP gene expression [195] and VIP release [196].

Unlike CGRP, which is a large molecule peptide and is thought to not penetrate the blood–brain barrier in significant concentrations, PACAP has a transporter pump offering the ability to cross the blood–brain barrier [197]. This may explain why premonitory symptoms are less readily provoked by CGRP compared to NTG and PACAP [80,101,136,198]. PACAP is heavily expressed in the hypothalamus [199], and this area of the brain is thought to be crucial in mediating premonitory symptoms [13,61,63,200].

A shared mechanism between CGRP and PACAP in migraine biology is the possible role of both peptides in mediating photophobia. These mechanisms are likely to be distinct, given that both can trigger light-aversive behaviours in animal models, but CGRP-mediated light aversion could only be inhibited by a CGRP monoclonal antibody and not a PACAP one, and vice versa [201]. PACAP can also provoke periorbital allodynia in animal models, and this can be reversed with a PACAP receptor antagonist [202]. Other mouse models have also suggested distinct roles of CGRP and PACAP in mediating migraine-like behaviours, such as hypersensitivity as a correlate for allodynia [203]. Mice pre-treated with an anti-CGRP antibody and in RAMP-1 knockout mice lacking CGRP receptors could still display allodynic behaviours in response to PACAP, but NTG-mediated allodynia could be prevented by anti-CGRP antibodies and in the RAMP-1 knockout mice. NTG and CGRP, therefore, are likely to act via shared mechanisms distinct from PACAP in mediating sensory sensitivities, but the interaction of these molecules in the wider migraine phenotype remains unclear. A role of PACAP in intracellular signalling pathways, some shared with CGRP given the co-expression of both peptides in many brain regions involved in migraine, like those via cAMP, is also likely [204].

### 8.2. Clinical Evidence for PACAP in Migraine

PACAP, particularly PACAP-38, which makes up around 90% of all PACAP (and the remainder is PACAP-27), is another neuropeptide, which, like CGRP, is released during acute migraine in both experimental and clinical settings, and blood levels are reduced following sumatriptan administration [30,205]. There is also a suggestion that blood levels of PACAP fluctuate in a dynamic fashion during different phases of migraine and may actually be lower in patients with migraine compared to healthy controls interictally but at a peak ictally [29]. Interestingly, PACAP levels are elevated in migraine attacks, but VIP levels are only elevated if there are CAS associated [23], and both are elevated in the cranial circulation in cluster headache [206], a condition in which CAS are necessary for diagnosis. This suggests that PACAP and VIP are both involved in the mediation of CAS, with PACAP having additional roles in trigeminovascular nociceptive processing.

PACAP38 can also trigger migraine-like attacks [31] and premonitory symptoms [136] when infused into patients with migraine. PACAP-triggered migraine can be prevented with pre-medication with sumatriptan [207]. Human imaging studies have shown that similarly to NTG and CGRP, PACAP is a vasodilator of the extracranial vasculature and does not affect intracranial arteries [36,208], and PACAP-triggered migraine causes widespread functional brain network changes [209]. There is a suggestion that PACAP27 can also trigger migraine [210], with further work in this area warranted.

### 8.3. Therapeutic Scope

The suggestion of a central PAC1-mediated mechanism in migraine suggested by initial rodent studies has been further interrogated using a rodent-specific PAC1 receptor antibody. The antibody was able to reduce trigeminocervical complex firing in response to stimulation, which was mediated via its binding at the trigeminal and sphenopalatine ganglia without central binding [32]. This study supported the role of targeting the PAC1 receptor in migraine therapeutics. Recently, a phase 2 clinical trial conducted by Lundbeck has released exciting positive results of a PACAP monoclonal antibody acting against the PACAP ligand in human migraine prevention (Lu AG09222) [33], and phase 3 studies are likely to follow to further investigate this effect. A recent healthy control study suggested that the antibody was able to reduce PACAP and VIP-induced facial vasodilatation and mild headaches in this patient group [211].

Targeting the PACAP pathway, as a neuropeptide pathway distinct from CGRP, holds therapeutic promise in migraine therapeutics going forwards.

## 9. Vasoactive Intestinal Peptide (VIP)

### 9.1. Preclinical Evidence for the Role of VIP in Migraine

Parasympathetic cell bodies supplying the perivascular nerves of the cranial circulation are found in the otic and sphenopalatine ganglia and contain VIP/PACAP and NOS, whilst PACAP is also found in trigeminal ganglion without VIP co-expression [212]. In animal models, stimulation of the superior sagittal sinus as an experimental migraine model causes raised external jugular VIP levels [106], and locus coeruleus stimulation could increase cranial blood flow by a mechanism antagonised by VIP polyclonal antibodies [213]. Immunohistochemical studies subsequently demonstrated VIP-ergic fibres in central nervous system areas important in migraine, such as the PAG and NRM [191], although, interestingly, not in the TNC or C1 and C2 [192]. A study examining neuropeptide and NOS co-localisation and interacting effects found that increasing PACAP concentrations caused CGRP release in the TNC, and there was no effect of VIP nor of a PAC1 agonist or antagonist on CGRP levels [214]. PACAP and VIP had no effect on NOS activity, and CGRP and PACAP shared co-localisation in the TNC and trigeminal ganglion, and PACAP and nNOS did so in the trigeminal ganglion [214]. This study therefore suggested that CGRP and NOS mechanisms may interact in migraine, PACAP mechanisms may be somewhat distinct, and VIP likely also does not interact with the CGRP and NOS systems.

Similarly to CGRP, VIP can induce photophobia in a rodent model [215], but it failed to induce periorbital allodynia in a mouse model [202]. In a rat model of migraine, using unilateral sympathectomy, VIP could reduce sympathectomy-induced raised dural NO levels [216]. Using a similar model, VIP reduced mast cell numbers and immunoreactivity in the ipsilateral trigeminal nucleus, which the authors hypothesised suggests the role of VIP as a modulator of neurogenic dural inflammation [217].

### 9.2. Clinical Evidence for the Role of VIP in Migraine

VIP is a potent vasodilatory neuropeptide that has dominant effects on the cranial and extracranial vasculature via parasympathetic mechanisms [23]. VIP levels have been shown to be elevated in interictal episodic and chronic migraine patients and to correlate with clinical CAS associated with migraine [133,218]. These levels normalise in saliva [219] and in external jugular blood [220] with headache abortion following triptan therapy. Serum VIP levels can also predict clinical outcomes following botulinum toxin therapy in migraine [221] and may therefore be an interesting therapeutic biomarker in migraine.

Whilst initially, VIP infusion was thought to not trigger migraine in humans despite causing extracranial arterial dilatation [34,36], nor did it cause significant vasodilatory headache in healthy controls [222], it was thought to be involved in CAS mediation in the rarer trigeminal autonomic cephalalgia (TAC) headaches [206] and in migraine [218,223]. There is, however, recent evidence that VIP, when administered as a prolonged infusion, may trigger migraine-like attacks in those with migraine [35] and delayed headaches in healthy controls [224]. Given that CAS are increasingly recognised in migraine [225] and can present in the premonitory phase before headache [80], combined with emerging evidence that VIP may, in fact, be involved in the headache phase of migraine, VIP may emerge as a potential therapeutic target in migraine again in the future. In the SPG, VIP is co-expressed with CGRP and PACAP [27,112], and this may provide an interaction between the sensory and parasympathetic systems in migraine.

### 9.3. Therapeutic Scope

The recent demonstration that VIP can, in fact, trigger migraine in those with underlying migraine [35], as well as other migraine-related behaviours in animal models [226], suggests that the VPAC1 and VPAC2 receptors may also hold therapeutic potential as migraine targets for the future.

## 10. Neuropeptide Y (NPY)

### 10.1. Preclinical Evidence for the Role of NPY in Migraine

NPY, a neuropeptide involved in the sympathetic nervous system, acts as a vasoconstrictor and interacts with the orexinergic pathways in the hypothalamus, thereby modulating several physiological processes such as sleep and feeding (promotion of feeding) [227]. It is co-localised with noradrenaline in sympathetic nerve terminals [191] and may therefore be involved in the adrenergic influence on the TNC via the LC in the pons. NPY is also located in the intra- and extracranial vasculature, where it plays a role in cerebral blood flow regulation [228]. Given the links between these physiological mechanisms and migraine, NPY has been of interest in migraine. NPY binds to several receptors, including NPY Y1 and NPY Y2 receptors, which are located within the trigeminal sensory system in the trigeminal ganglion and TNC [192], as well as in the hypothalamus and limbic brain areas involved in behavioural, emotional, and homeostatic regulation [229]. NPY injection into the hypothalamus promotes feeding [230], and this response is modulated via orexin A, which activates it [231], and leptin, which inhibits it [232]. NPY is localised in orexinergic hypothalamic neurons that project to several pain-modulating areas, such as the PAG [233], and is involved in nociception [234]. Orexins have been implicated in migraine biology [235,236,237,238], and orexin A reduces TCC firing in a rodent model of migraine [238] and also promotes NPY release [239], so these neuropeptides are likely to anatomically and functionally interact in physiological and perhaps migraine pain mechanisms.

In a rodent model, NPY reduced neuronal activity in the TCC, which was reduced by an NPY Y1 agonist, suggesting that NPY may be involved in trigeminovascular processing via an NPY Y1 mechanism [240]. Subsequently, a rat model of migraine using repeated electrical stimulation of the trigeminal ganglion revealed increased NPY levels (as well as CGRP, PACAP, and VIP) in both trigeminal ganglion and blood [241]. In an NTG mouse model of migraine, NPY Y1 activation reduced NTG-provoked allodynic and anxiety behaviours via the habenula, suggesting that the NPY Y1 receptor is involved in analgesic and anxiolytic effects following NTG exposure [242]. NPY Y1 deficient mice seem to display mild hyperinsulinaemia and obesity, and this could be relevant in the interactions between glucose and insulin metabolism and migraine [243]. A rodent study also demonstrated supportive evidence for the role of feeding hormones, such as insulin, leptin, and glucagon, on trigeminovascular sensory processing, thus supporting the interaction between sensory, metabolic, and homeostatic systems in migraine [244].

NPY has differing effects on sleep patterns in rodent models, depending on where it is administered [245,246]. It is also involved in stress responses via the hypothalamic–pituitary axis [247], and via its role in nociception as well as the physiological processes of feeding, sleep, and stress, may be involved in migraine biology.

### 10.2. Clinical Evidence for the Role of NPY in Migraine

Clinical studies in patients with migraine have revealed inconclusive results regarding blood NPY levels during attacks [23,248] and interictally [249,250,251], although further larger studies on patients not on migraine prevention are likely warranted. CSF NPY immunoreactivity has been demonstrated to be higher ictally in patients with migraine compared to controls in one study [252], although this study was in contrast to a former one which did not find altered CSF NPY reactivity in suboccipital CSF [251]. Specific NPY antagonism has not been tried in migraine, but a dual orexin antagonist failed in a clinical trial of migraine [253], although this was a non-specific drug dosed once at night. More targeted approaches and different dosing schedules may yield different results.

The interaction between feeding and migraine, in that some foods are considered migraine triggers and migraine attacks can cause altered feeding (cravings, hyperphagia, or anorexia) [254], and the link between obesity and migraine [255] and between type 2 diabetes and migraine, all implicate the interaction between feeding hormones such as NPY in migraine biology [256]. A study found that insulin resistance amongst non-obese premenopausal women with chronic migraine could be mediated by NPY, as fasting levels were found to be elevated [257], demonstrating a possible link between migraine and these other disorders via feeding pathways. In addition, NPY has roles in sleep and shortens sleep latency in humans [258] and in response to stress and mood [247], providing possible links between migraine and altered physiological processes that are commonly reported by patients [259].

### 10.3. Therapeutic Scope

Whilst no specific NPY agonists have been trialled clinically in migraine, NPY Y1 agonism may hold therapeutic promise as a future target. Specific targeting within the orexinergic pathway may also hold similar promise and provide an opportunity to target more than the pain phase of the attack.

Some of the neuropeptides that have been implicated in migraine are summarised in Table 1.

## 11. Conclusions

**Figure 1:**
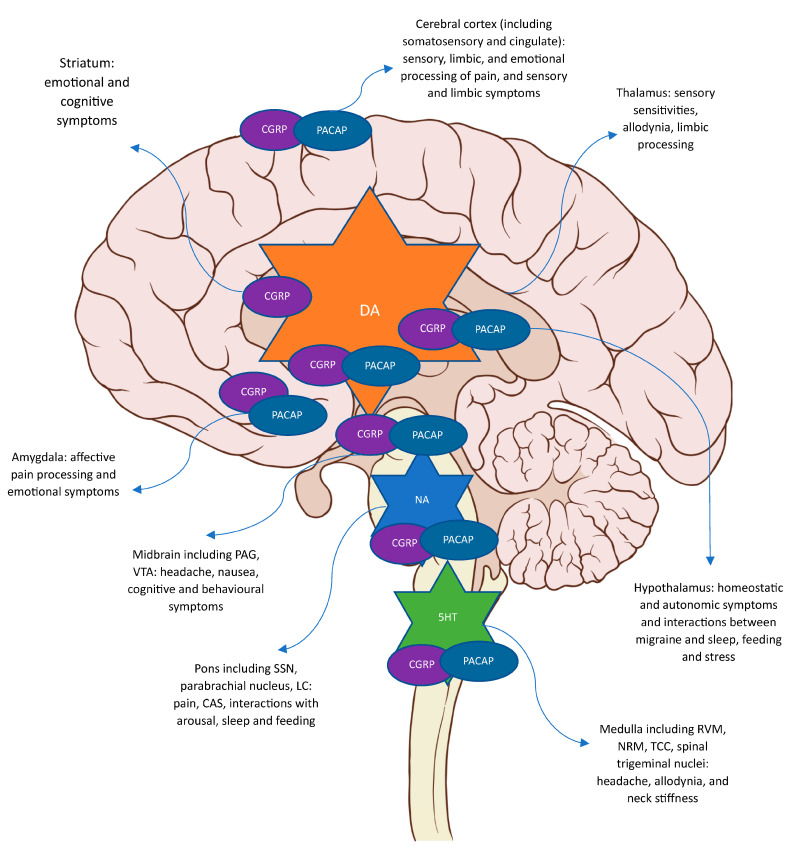
Summary of the interaction between monoaminergic and peptidergic neurotransmission centrally via areas of interest in migraine biology and the symptom correlates for each area (dural vasculature, trigeminal ganglion, and SPG are not included). VIP is likely involved in peripheral mechanisms of dural vasodilatation and the parasympathetic reflex via the dural vasculature, SSN and SPG. DA; dopamine, NA; noradrenaline, and 5HT; serotonin. Free to use sagittal brain image https://upload.wikimedia.org/wikipedia/commons/a/a0/Brain_human_sagittal_section.svgby, Patrick J. Lynch, medical illustrator, CC BY 2.5 <https://creativecommons.org/licenses/by/2.5>, via Wikimedia Commons. The authors have annotated the figure for the purpose of this article.

Migraine is a complex disorder of altered brain sensory, limbic, and homeostatic processing and involves the anatomical and functional interactions between pathways involving nociception and other physiological processes, such as those modulating sleep, behaviour, feeding, and mood. Several brain areas are affected in mediating the heterogeneous clinical phenotype and involve several monoaminergic diencephalic and brainstem nuclei, even before pain onset in the premonitory phase, when these areas act as feasible neural substrates for premonitory symptoms [1]. There is also preclinical and clinical evidence for the role of these neurotransmitters in trigeminal pain perception [14]. Various neurotransmitters may be involved via important brain nuclei and regions and support the interplay between nociceptive and physiological mechanisms in migraine. Some of these are peptidergic and involve vasodilatory neuropeptides such as CGRP, PACAP, and VIP, the vasoconstrictive NPY, and other neuropeptides such as the orexins and somatostatin [265]. Others are monoaminergic and exert their actions via similar brain areas and involve dopamine, serotonin, adrenaline, and noradrenaline. NO is also involved in vasodilatation and in interacting with CGRP transmission. Neuropeptide release in the periphery, at the level of the dural vasculature, is likely involved in vasodilatation and peripheral sensitisation via CGRP, PACAP, and VIP. PACAP and VIP are likely involved in CAS mediation, but given these can present before headache in both migraine [80] and cluster headache [266,267], activation of this reflex does not require headache. PACAP and NOS may be involved in attack initiation, given their ability to trigger premonitory symptoms [80,101,136,198]. Central effects of CGRP and PACAP may be involved in pain transmission at the level of the TCC and in central sensitisation mechanisms. The role of these neuropeptides in other symptoms of migraine, like premonitory symptoms, remains to be further investigated. The interactions between monoaminergic and peptidergic neurotransmission centrally are likely the basis of the molecular circuitry of migraine, both before and during headaches. The interaction between monoaminergic and peptidergic neurotransmission via brain areas of interest and which symptoms may be displayed by these are summarised in Figure 1.

Identification of these implicated molecules has led to the exciting translation of bench-to-bedside research over recent years, with the emergence of peptide-targeted therapies in migraine clinical practice since the triptan era in the 1990s. Other peptide targets hold promise for the future. Much remains to be learned about the molecular and neurochemical basis of migraine, but it is clear that there is unlikely to be a ‘one shoe fits all’ therapeutic approach that will serve all sufferers. Despite extracranial vasodilatation being a feature of migraine and many of the implicated neuropeptides being vasodilatory, vasoconstriction is neither necessary nor required for pain abortion. The development of different targeted strategies is, therefore, key to improving the lives of those underserved by current therapies, and dissecting molecular circuitry, receptor systems, and non-vascular targets is likely to be the best way forward in the future.

## 12. Future Directions

Designated targeting of the PAC1, NPY Y1, and OX1 receptors [14] and of nNOS may form exciting therapeutic options for migraine. An understanding of different receptor subtypes and their roles will allow further development of specific agents which lack the adverse effects of broad receptor targets. The ability to treat headaches as well as other migraine-associated symptoms via neuropeptides that link headaches and other physiological systems is attractive to both physicians and patients to ultimately limit migraine-related burden. The role of PACAP and NOS in premonitory symptoms remains to be elucidated, and the treatment of the migraine attack before headaches with targeted treatments against these systems may hold promise in treating associated symptoms and in preventing pain onset. This has already been demonstrated in early studies of ubrogepant [166,167], despite CGRP previously showing a lower affinity for triggering premonitory symptoms compared to NTG and PACAP [136]. The potential ability to treat the migraine attack before the headache has even started is a unique opportunity in migraine, and the role of targeting NOS and peptidergic therapies in this signals the start of an exciting era in migraine therapeutics, which is only likely to advance with time and increased understanding of these pathways, mechanisms, and interactions. 

## Figures and Tables

**Table 1:** Some of the neuropeptides that have been implicated in migraine biology and the possible role of targeting them in migraine therapeutics.

Neuropeptide	Role in Migraine	Role in Migraine Therapeutics
CGRP	Sensitisation, vasodilatation, mast cell degranulation, CSD, circadian variability to migraine, photophobia, allodynia, and interactions with NOS	Small molecule CGRP antagonists (*gepants*) for attack abortion and rimegepant for both acute and preventive effects (oral and nasal formulations) Monoclonal antibodies against CGRP ligand or canonical receptor for migraine prevention (subcutaneous and intravenous formulations)
PACAP	Vasodilatation, photophobia, allodynia, sensitisation, and CASs	PAC1 and PACAP receptor antibodies hold potential
VIP	Vasodilatation, CASs, mast cell regulation, and interaction between sensory and parasympathetic systems	VPAC1 and VPAC2 receptors may hold potential in the future
Neuropeptide Y	Regulation of sleep, promotion of feeding, stress responses, anxiety, allodynia, and interaction between pain and physiological processes	NPY Y1 agonism may hold potential in the future
Agouti-related peptide and proopiomelanocortin (POMC) and cocaine and amphetamine-related transcript (CART)	Regulation of feeding, with opposing signalling effects in response to leptin, hypothalamic projections regulating energy balance homeostasis, and links between migraine and feeding and obesity and diabetes	Nil as yet, but these peptides alter appetite and may be implicated in the links between migraine and disordered feeding (and there may be a role of the ventral tegmental area in both migraine headache and modulating food craving) [260,261]
Orexins A and B	Sleep regulation, glucose metabolism, and possible preclinical role in headache [236,237]	Specific Ox1 antagonism may hold therapeutic promise in the future [253]
Leptin	Appetite suppression and possible link to raised BMI in migraine [262], preclinical evidence for a possible role in headache [244]	Targeting the interactions between migraine and impaired metabolic homeostasis may hold future therapeutic promise
Substance P and neurokinin A	Vasodilatation, plasma protein extravasation, mast cell degranulation, and platelet aggregation	Neurokinin 1 inhibition and plasma protein extravasation inhibitors have failed in clinical trials of migraine [263,264]

## Data Availability

Not applicable.

## References

[1] Karsan N., Goadsby P.J. (2018). Biological insights from the premonitory symptoms of migraine. Nat. Rev. Neurol..

[2] Viana M., Sances G., Linde M., Ghiotto N., Guaschino E., Allena M., Terrazzino S., Nappi G., Goadsby P.J., Tassorelli C. (2017). Clinical features of migraine aura: Results from a prospective diary-aided study. Cephalalgia.

[3] Polderman T.J., Benyamin B., de Leeuw C.A., Sullivan P.F., van Bochoven A., Visscher P.M., Posthuma D. (2015). Meta-analysis of the heritability of human traits based on fifty years of twin studies. Nat. Genet..

[4] Gormley P., Anttila V., Winsvold B.S., Palta P., Esko T., Pers T.H., Farh K.H., Cuenca-Leon E., Muona M., Furlotte N.A. (2016). Meta-analysis of 375,000 individuals identifies 38 susceptibility loci for migraine. Nat. Genet..

[5] Anttila V., Stefansson H., Kallela M., Todt U., Terwindt G.M., Calafato M.S., Nyholt D.R., Dimas A.S., Freilinger T., Müller-Myhsok B. (2010). Genome-wide association study of migraine implicates a common susceptibility variant on 8q22.1. Nat. Genet..

[6] Chasman D.I., Schürks M., Anttila V., de Vries B., Schminke U., Launer L.J., Terwindt G.M., van den Maagdenberg A.M., Fendrich K., Völzke H. (2011). Genome-wide association study reveals three susceptibility loci for common migraine in the general population. Nat. Genet..

[7] Choquet H., Yin J., Jacobson A.S., Horton B.H., Hoffmann T.J., Jorgenson E., Avins A.L., Pressman A.R. (2021). New and sex-specific migraine susceptibility loci identified from a multiethnic genome-wide meta-analysis. Commun. Biol..

[8] Hautakangas H., Winsvold B.S., Ruotsalainen S.E., Bjornsdottir G., Harder A.V.E., Kogelman L.J.A., Thomas L.F., Noordam R., Benner C., Gormley P. (2022). Genome-wide analysis of 102,084 migraine cases identifies 123 risk loci and subtype-specific risk alleles. Nat. Genet..

[9] Anttila V., Winsvold B.S., Gormley P., Kurth T., Bettella F., McMahon G., Kallela M., Malik R., de Vries B., Terwindt G. (2013). Genome-wide meta-analysis identifies new susceptibility loci for migraine. Nat. Genet..

[10] Russell M.B., Rasmussen B.K., Fenger K., Olesen J. (1996). Migraine without aura and migraine with aura are distinct clinical entities: A study of four hundred and eighty-four male and female migraineurs from the general population. Cephalalgia.

[11] Grangeon L., Lange K.S., Waliszewska-Prosół M., Onan D., Marschollek K., Wiels W., Mikulenka P., Farham F., Gollion C., Ducros A. (2023). Genetics of migraine: Where are we now?. J. Headache Pain.

[12] Ferrari M.D., Goadsby P.J., Burstein R., Kurth T., Ayata C., Charles A., Ashina M., van den Maagdenberg A., Dodick D.W. (2022). Migraine. Nat. Rev. Dis. Primers.

[13] Karsan N., Bose R.P., O’Daly O., Zelaya F., Goadsby P.J. (2023). Regional cerebral perfusion during the premonitory phase of triggered migraine: A double-blind randomized placebo-controlled functional imaging study using pseudo-continuous arterial spin labeling. Headache.

[14] Goadsby P.J., Holland P.R., Martins-Oliveira M., Hoffmann J., Schankin C., Akerman S. (2017). Pathophysiology of Migraine: A Disorder of Sensory Processing. Physiol. Rev..

[15] Headache Classification Committee of the International Headache Society (IHS) (2018). The International Classification of Headache Disorders, 3rd edition. Cephalalgia.

[16] Viana M., Linde M., Sances G., Ghiotto N., Guaschino E., Allena M., Terrazzino S., Nappi G., Goadsby P.J., Tassorelli C. (2016). Migraine aura symptoms: Duration, succession and temporal relationship to headache. Cephalalgia.

[17] Viana M., Sances G., Linde M., Nappi G., Khaliq F., Goadsby P.J., Tassorelli C. (2018). Prolonged migraine aura: New insights from a prospective diary-aided study. J. Headache Pain.

[18] Schankin C.J., Viana M., Goadsby P.J. (2017). Persistent and Repetitive Visual Disturbances in Migraine: A Review. Headache.

[19] Rasmussen B.K., Olesen J. (1992). Migraine with aura and migraine without aura: An epidemiological study. Cephalalgia.

[20] Ashina M., Hansen J.M., á Dunga B.O., Olesen J. (2017). Human models of migraine—Short-term pain for long-term gain. Nat. Rev. Neurol..

[21] Iversen H.K., Olesen J., Tfelt-Hansen P. (1989). Intravenous nitroglycerin as an experimental model of vascular headache. Basic characteristics. Pain.

[22] Goadsby P.J., Edvinsson L., Ekman R. (1988). Release of vasoactive peptides in the extracerebral circulation of humans and the cat during activation of the trigeminovascular system. Ann. Neurol..

[23] Goadsby P.J., Edvinsson L., Ekman R. (1990). Vasoactive peptide release in the extracerebral circulation of humans during migraine headache. Ann. Neurol..

[24] Goadsby P.J., Edvinsson L. (1993). The trigeminovascular system and migraine: Studies characterising cerebrovascular and neuropeptide changes seen in humans and cats. Ann. Neurol..

[25] Lassen L.H., Haderslev P.A., Jacobsen V.B., Iversen H.K., Sperling B., Olesen J. (2002). CGRP may play a causative role in migraine. Cephalalgia.

[26] Edvinsson L., Haanes K.A., Warfvinge K., Krause D.N. (2018). CGRP as the target of new migraine therapies—Successful translation from bench to clinic. Nat. Rev. Neurol..

[27] Steinberg A., Frederiksen S.D., Blixt F.W., Warfvinge K., Edvinsson L. (2016). Expression of messenger molecules and receptors in rat and human sphenopalatine ganglion indicating therapeutic targets. J. Headache Pain.

[28] Akerman S., Goadsby P.J. (2015). Neuronal PAC1 receptors mediate delayed activation and sensitization of trigeminocervical neurons: Relevance to migraine. Sci. Transl. Med..

[29] Tuka B., Helyes Z., Markovics A., Bagoly T., Szolcsanyi J., Szabo N., Toth E., Kincses Z.T., Vecsei L., Tajti J. (2013). Alterations in PACAP-38-like immunoreactivity in the plasma during ictal and interictal periods of migraine patients. Cephalalgia.

[30] Zagami A.S., Edvinsson L., Goadsby P.J. (2014). Pituitary adenylate cyclase activating polypeptide and migraine. Ann. Clin. Transl. Neurol..

[31] Schytz H.W., Birk S., Wienecke T., Kruuse C., Olesen J., Ashina M. (2009). PACAP38 induces migraine-like attacks in patients with migraine without aura. Brain J. Neurol..

[32] Hoffmann J., Miller S., Martins-Oliveira M., Akerman S., Supronsinchai W., Sun H., Shi L., Wang J., Zhu D., Lehto S. (2020). PAC1 receptor blockade reduces central nociceptive activity: New approach for primary headache?. Pain.

[33] Lundbeck Lundbeck Announces Positive Phase II Proof of Concept Results with Lu AG09222 in Migraine Prevention. https://news.cision.com/h--lundbeck-a-s/r/lundbeck-announces-positive-phase-ii-proof-of-concept-results-with-lu-ag09222-in-migraine-prevention,c3754245.

[34] Rahmann A., Wienecke T., Hansen J.M., Fahrenkrug J., Olesen J., Ashina M. (2008). Vasoactive intestinal peptide causes marked cephalic vasodilation, but does not induce migraine. Cephalalgia.

[35] Pellesi L., Al-Karagholi M.A., De Icco R., Coskun H., Elbahi F.A., Lopez-Lopez C., Snellman J., Hannibal J., Amin F.M., Ashina M. (2021). Effect of Vasoactive Intestinal Polypeptide on Development of Migraine Headaches: A Randomized Clinical Trial. JAMA Netw. Open.

[36] Amin F.M., Hougaard A., Schytz H.W., Asghar M.S., Lundholm E., Parvaiz A.I., de Koning P.J., Andersen M.R., Larsson H.B., Fahrenkrug J. (2014). Investigation of the pathophysiological mechanisms of migraine attacks induced by pituitary adenylate cyclase-activating polypeptide-38. Brain J. Neurol..

[37] Asghar M.S. (2012). Effects of glyceryl trinitrate and calcitonin gene-related peptide on BOLD signal and arterial diameter: Methodological studies by fMRI and MRA. Dan. Med. J..

[38] Amin F.M., Asghar M.S., Hougaard A., Hansen A.E., Larsen V.A., de Koning P.J., Larsson H.B., Olesen J., Ashina M. (2013). Magnetic resonance angiography of intracranial and extracranial arteries in patients with spontaneous migraine without aura: A cross-sectional study. Lancet Neurol..

[39] Christiansen I., Thomsen L.L., Daugaard D., Ulrich V., Olesen J. (1999). Glyceryl trinitrate induces attacks of migraine without aura in sufferers of migraine with aura. Cephalalgia.

[40] Hansen J.M., Hauge A.W., Olesen J., Ashina M. (2010). Calcitonin gene-related peptide triggers migraine-like attacks in patients with migraine with aura. Cephalalgia.

[41] Hansen J.M., Thomsen L.L., Olesen J., Ashina M. (2008). Familial hemiplegic migraine type 1 shows no hypersensitivity to nitric oxide. Cephalalgia.

[42] Hansen J.M., Thomsen L.L., Olesen J., Ashina M. (2008). Calcitonin gene-related peptide does not cause the familial hemiplegic migraine phenotype. Neurology.

[43] Hansen J.M., Thomsen L.L., Marconi R., Casari G., Olesen J., Ashina M. (2008). Familial hemiplegic migraine type 2 does not share hypersensitivity to nitric oxide with common types of migraine. Cephalalgia.

[44] Hansen J.M., Thomsen L.L., Olesen J., Ashina M. (2011). Calcitonin gene-related peptide does not cause migraine attacks in patients with familial hemiplegic migraine. Headache.

[45] Ghanizada H., Al-Karagholi M.A., Walker C.S., Arngrim N., Rees T., Petersen J., Siow A., Mørch-Rasmussen M., Tan S., O’Carroll S.J. (2021). Amylin Analog Pramlintide Induces Migraine-like Attacks in Patients. Ann. Neurol..

[46] Ghanizada H., Al-Karagholi M.A., Arngrim N., Mørch-Rasmussen M., Walker C.S., Hay D.L., Ashina M. (2021). Effect of Adrenomedullin on Migraine-Like Attacks in Patients with Migraine: A Randomized Crossover Study. Neurology.

[47] Al-Karagholi M.A., Ghanizada H., Nielsen C.A.W., Hougaard A., Ashina M. (2021). Opening of ATP sensitive potassium channels causes migraine attacks with aura. Brain J. Neurol..

[48] Al-Karagholi M.A., Hansen J.M., Guo S., Olesen J., Ashina M. (2019). Opening of ATP-sensitive potassium channels causes migraine attacks: A new target for the treatment of migraine. Brain J. Neurol..

[49] Weiller C., May A., Limmroth V., Juptner M., Kaube H., Schayck R.V., Coenen H.H., Diener H.C. (1995). Brain stem activation in spontaneous human migraine attacks. Nat. Med..

[50] Bahra A., Matharu M.S., Buchel C., Frackowiak R.S., Goadsby P.J. (2001). Brainstem activation specific to migraine headache. Lancet.

[51] Denuelle M., Fabre N., Payoux P., Chollet F., Geraud G. (2007). Hypothalamic activation in spontaneous migraine attacks. Headache.

[52] Afridi S.K., Matharu M.S., Lee L., Kaube H., Friston K.J., Frackowiak R.S., Goadsby P.J. (2005). A PET study exploring the laterality of brainstem activation in migraine using glyceryl trinitrate. Brain J. Neurol..

[53] Moragues N., Ciofi P., Lafon P., Odessa M.F., Tramu G., Garret M. (2000). cDNA cloning and expression of a gamma-aminobutyric acid A receptor epsilon-subunit in rat brain. Eur. J. Neurosci..

[54] Bonnert T.P., McKernan R.M., Farrar S., le Bourdellès B., Heavens R.P., Smith D.W., Hewson L., Rigby M.R., Sirinathsinghji D.J., Brown N. (1999). theta, a novel gamma-aminobutyric acid type A receptor subunit. Proc. Natl. Acad. Sci. USA.

[55] Ray B.S., Wolff H.G. (1940). Experimental studies on headache. Pain sensitive structures of the head and their significance in headache. Arch. Surg..

[56] Uddman R., Goadsby P.J., Jansen I., Edvinsson L. (1993). PACAP, a VIP-like peptide: Immunohistochemical localization and effect upon cat pial arteries and cerebral blood flow. J. Cereb. Blood Flow Metab. Off. J. Int. Soc. Cereb. Blood Flow Metab..

[57] Uddman R., Edvinsson L., Ekman R., Kingman T., McCulloch J. (1985). Innervation of the feline cerebral vasculature by nerve fibers containing calcitonin gene-related peptide: Trigeminal origin and co-existence with substance P. Neurosci. Lett..

[58] Edvinsson L., Brodin E., Jansen I., Uddman R. (1988). Neurokinin A in cerebral vessels: Characterization, localization and effects in vitro. Regul. Pept..

[59] Akerman S., Holland P.R., Goadsby P.J. (2011). Diencephalic and brainstem mechanisms in migraine. Nat. Rev. Neurol..

[60] Hoffmann J., Baca S.M., Akerman S. (2019). Neurovascular mechanisms of migraine and cluster headache. J. Cereb. Blood Flow Metab. Off. J. Int. Soc. Cereb. Blood Flow Metab..

[61] Maniyar F.H., Sprenger T., Monteith T., Schankin C., Goadsby P.J. (2014). Brain activations in the premonitory phase of nitroglycerin-triggered migraine attacks. Brain J. Neurol..

[62] Karsan N., Bose P.R., O’Daly O., Zelaya F.O., Goadsby P.J. (2020). Alterations in Functional Connectivity During Different Phases of the Triggered Migraine Attack. Headache.

[63] Schulte L.H., Menz M.M., Haaker J., May A. (2020). The migraineur’s brain networks: Continuous resting state fMRI over 30 days. Cephalalgia.

[64] Schulte L.H., May A. (2016). The migraine generator revisited: Continuous scanning of the migraine cycle over 30 days and three spontaneous attacks. Brain J. Neurol..

[65] Giffin N.J., Ruggiero L., Lipton R.B., Silberstein S.D., Tvedskov J.F., Olesen J., Altman J., Goadsby P.J., Macrae A. (2003). Premonitory symptoms in migraine: An electronic diary study. Neurology.

[66] Tassorelli C., Greco R., Morocutti A., Costa A., Nappi G. (2001). Nitric oxide-induced neuronal activation in the central nervous system as an animal model of migraine: Mechanisms and mediators. Funct. Neurol..

[67] Tassorelli C., Joseph S.A. (1995). Systemic nitroglycerin induces fos immunoreactivity in brainstem and forebrain structures of the rat. Brain Res..

[68] Akerman S., Karsan N., Bose P., Hoffmann J.R., Holland P.R., Romero-Reyes M., Goadsby P.J. (2019). Nitroglycerine triggers triptan-responsive cranial allodynia and trigeminal neuronal hypersensitivity. Brain J. Neurol..

[69] Zhang X., Kainz V., Zhao J., Strassman A.M., Levy D. (2013). Vascular extracellular signal-regulated kinase mediates migraine-related sensitization of meningeal nociceptors. Ann. Neurol..

[70] Tassorelli C., Costa A., Blandini F., Joseph S.A., Nappi G. (2000). Effect of nitric oxide donors on the central nervous system--nitroglycerin studies in the rat. Funct. Neurol..

[71] Lambert G.A., Donaldson C., Boers P.M., Zagami A.S. (2000). Activation of trigeminovascular neurons by glyceryl trinitrate. Brain Res..

[72] Pardutz A., Krizbai I., Multon S., Vecsei L., Schoenen J. (2000). Systemic nitroglycerin increases nNOS levels in rat trigeminal nucleus caudalis. Neuroreport.

[73] Akerman S., Romero-Reyes M., Karsan N., Bose P., Hoffmann J.R., Holland P.R., Goadsby P.J. (2021). Therapeutic targeting of nitroglycerin-mediated trigeminovascular neuronal hypersensitivity predicts clinical outcomes of migraine abortives. Pain.

[74] Karsan N., Goadsby P.J. (2022). New Oral Drugs for Migraine. CNS Drugs.

[75] Hoskin K.L., Bulmer D.C., Goadsby P.J. (1999). Fos expression in the trigeminocervical complex of the cat after stimulation of the superior sagittal sinus is reduced by L-NAME. Neurosci. Lett..

[76] Pourrahimi A.M., Abbasnejad M., Raoof M., Esmaeili-Mahani S., Kooshki R. (2021). The involvement of orexin 1 and cannabinoid 1 receptors within the ventrolateral periaqueductal gray matter in the modulation of migraine-induced anxiety and social behavior deficits of rats. Peptides.

[77] Pradhan A.A., Smith M.L., McGuire B., Tarash I., Evans C.J., Charles A. (2014). Characterization of a novel model of chronic migraine. Pain.

[78] Koulchitsky S., Fischer M.J., De Col R., Schlechtweg P.M., Messlinger K. (2004). Biphasic response to nitric oxide of spinal trigeminal neurons with meningeal input in rat--possible implications for the pathophysiology of headaches. J. Neurophysiol..

[79] Fischer M.J., Koulchitsky S., Messlinger K. (2005). The nonpeptide calcitonin gene-related peptide receptor antagonist BIBN4096BS lowers the activity of neurons with meningeal input in the rat spinal trigeminal nucleus. J. Neurosci. Off. J. Soc. Neurosci..

[80] Karsan N., Bose P.R., Thompson C., Newman J., Goadsby P.J. (2020). Headache and non-headache symptoms provoked by nitroglycerin in migraineurs: A human pharmacological triggering study. Cephalalgia.

[81] Miyamoto T., Dubin A.E., Petrus M.J., Patapoutian A. (2009). TRPV1 and TRPA1 mediate peripheral nitric oxide-induced nociception in mice. PLoS ONE.

[82] Chizh B., Palmer J., Lai R., Guillard F., Bullman J., Baines A., Napolitano A., Appleby J. (2009). A randomised, two-period cross-over study to investigate the efficacy of the Trpv1 antagonist SB-705498 in acute migraine. Eur. J. Pain.

[83] Messlinger K., Lennerz J.K., Eberhardt M., Fischer M.J. (2012). CGRP and NO in the trigeminal system: Mechanisms and role in headache generation. Headache.

[84] Akerman S., Williamson D.J., Kaube H., Goadsby P.J. (2002). Nitric oxide synthase inhibitors can antagonize neurogenic and calcitonin gene-related peptide induced dilation of dural meningeal vessels. Br. J. Pharmacol..

[85] Fletcher A., McLoone P., Bulpitt C. (1988). Quality of life on angina therapy: A randomised controlled trial of transdermal glyceryl trinitrate against placebo. Lancet.

[86] Sicuteri F., Del Bene E., Poggioni M., Bonazzi A. (1987). Unmasking latent dysnociception in healthy subjects. Headache.

[87] Ignarro L.J., Lippton H., Edwards J.C., Baricos W.H., Hyman A.L., Kadowitz P.J., Gruetter C.A. (1981). Mechanism of vascular smooth muscle relaxation by organic nitrates, nitrites, nitroprusside and nitric oxide: Evidence for the involvement of S-nitrosothiols as active intermediates. J. Pharmacol. Exp. Ther..

[88] Thomsen L.L., Iversen H.K., Brinck T.A., Olesen J. (1993). Arterial supersensitivity to nitric oxide (nitroglycerin) in migraine sufferers. Cephalalgia.

[89] Thomsen L.L., Kruuse C., Iversen H.K., Olesen J. (1994). A nitric oxide donor (nitroglycerin) triggers genuine migraine attacks. Eur. J. Neurol..

[90] Tassorelli C., Joseph S.A., Buzzi M.G., Nappi G. (1999). The effects on the central nervous system of nitroglycerin—Putative mechanisms and mediators. Prog. Neurobiol..

[91] Schoonman G.G., van der Grond J., Kortmann C., van der Geest R.J., Terwindt G.M., Ferrari M.D. (2008). Migraine headache is not associated with cerebral or meningeal vasodilatation—A 3T magnetic resonance angiography study. Brain J. Neurol..

[92] Iversen H.K., Olesen J. (1996). Headache induced by a nitric oxide donor (nitroglycerin) responds to sumatriptan. A human model for development of migraine drugs. Cephalalgia.

[93] Hoskin K.L., Kaube H., Goadsby P.J. (1996). Sumatriptan can inhibit trigeminal afferents by an exclusively neural mechanism. Brain J. Neurol..

[94] Lassen L.H., Ashina M., Christiansen I., Ulrich V., Grover R., Donaldson J., Olesen J. (1998). Nitric oxide synthase inhibition: A new principle in the treatment of migraine attacks. Cephalalgia.

[95] Palmer J.E., Guillard F.L., Laurijssens B.E., Wentz A.L., Dixon R.M. (2009). A randomised, single-blind, placebo-controlled, adaptive clinical trial of GW274150, a selective iNOS inhibitor, in the treatment of acute migraine. Cephalalgia.

[96] Høivik H.O., Laurijssens B.E., Harnisch L.O., Twomey C.K., Dixon R.M., Kirkham A.J., Williams P.M., Wentz A.L., Lunnon M.W. (2010). Lack of efficacy of the selective iNOS inhibitor GW274150 in prophylaxis of migraine headache. Cephalalgia.

[97] Hougaard A., Hauge A.W., Guo S., Tfelt-Hansen P. (2013). The nitric oxide synthase inhibitor and serotonin-receptor agonist NXN-188 during the aura phase of migraine with aura: A randomized, double-blind, placebo-controlled cross-over study. Scand. J. Pain.

[98] Sarchielli P., Alberti A., Codini M., Floridi A., Gallai V. (2000). Nitric oxide metabolites, prostaglandins and trigeminal vasoactive peptides in internal jugular vein blood during spontaneous migraine attacks. Cephalalgia.

[99] Juhasz G., Zsombok T., Modos E.A., Olajos S., Jakab B., Nemeth J., Szolcsanyi J., Vitrai J., Bagdy G. (2003). NO-induced migraine attack: Strong increase in plasma calcitonin gene-related peptide (CGRP) concentration and negative correlation with platelet serotonin release. Pain.

[100] Juhasz G., Zsombok T., Jakab B., Nemeth J., Szolcsanyi J., Bagdy G. (2005). Sumatriptan causes parallel decrease in plasma calcitonin gene-related peptide (CGRP) concentration and migraine headache during nitroglycerin induced migraine attack. Cephalalgia.

[101] Afridi S.K., Kaube H., Goadsby P.J. (2004). Glyceryl trinitrate triggers premonitory symptoms in migraineurs. Pain.

[102] Abrams J. (1985). Pharmacology of nitroglycerin and long-acting nitrates. Am. J. Cardiol..

[103] Bhatt D.K., Gupta S., Jansen-Olesen I., Andrews J.S., Olesen J. (2013). NXN-188, a selective nNOS inhibitor and a 5-HT1B/1D receptor agonist, inhibits CGRP release in preclinical migraine models. Cephalalgia.

[104] Medve R.A., Andrews J.S. (2009). Effects of fixed dose combination of nNOS inhibition and 5HT agonism on progression of migraine with and without aura. Cephalalgia.

[105] Reuter U., Bolay H., Jansen-Olesen I., Chiarugi A., Sanchez del Rio M., Letourneau R., Theoharides T.C., Waeber C., Moskowitz M.A. (2001). Delayed inflammation in rat meninges: Implications for migraine pathophysiology. Brain J. Neurol..

[106] Zagami A.S., Goadsby P.J., Edvinsson L. (1990). Stimulation of the superior sagittal sinus in the cat causes release of vasoactive peptides. Neuropeptides.

[107] Lennerz J.K., Ruhle V., Ceppa E.P., Neuhuber W.L., Bunnett N.W., Grady E.F., Messlinger K. (2008). Calcitonin receptor-like receptor (CLR), receptor activity-modifying protein 1 (RAMP1), and calcitonin gene-related peptide (CGRP) immunoreactivity in the rat trigeminovascular system: Differences between peripheral and central CGRP receptor distribution. J. Comp. Neurol..

[108] Eftekhari S., Gaspar R.C., Roberts R., Chen T.B., Zeng Z., Villarreal S., Edvinsson L., Salvatore C.A. (2016). Localization of CGRP receptor components and receptor binding sites in rhesus monkey brainstem: A detailed study using in situ hybridization, immunofluorescence, and autoradiography. J. Comp. Neurol..

[109] Eftekhari S., Edvinsson L. (2011). Calcitonin gene-related peptide (CGRP) and its receptor components in human and rat spinal trigeminal nucleus and spinal cord at C1-level. BMC Neurosci..

[110] Eftekhari S., Salvatore C.A., Calamari A., Kane S.A., Tajti J., Edvinsson L. (2010). Differential distribution of calcitonin gene-related peptide and its receptor components in the human trigeminal ganglion. Neuroscience.

[111] Csati A., Tajti J., Tuka B., Edvinsson L., Warfvinge K. (2012). Calcitonin gene-related peptide and its receptor components in the human sphenopalatine ganglion—Interaction with the sensory system. Brain Res..

[112] Csati A., Tajti J., Kuris A., Tuka B., Edvinsson L., Warfvinge K. (2012). Distribution of vasoactive intestinal peptide, pituitary adenylate cyclase-activating peptide, nitric oxide synthase, and their receptors in human and rat sphenopalatine ganglion. Neuroscience.

[113] Eftekhari S., Salvatore C.A., Johansson S., Chen T.B., Zeng Z., Edvinsson L. (2015). Localization of CGRP, CGRP receptor, PACAP and glutamate in trigeminal ganglion. Relation to the blood-brain barrier. Brain Res..

[114] Hou M., Kanje M., Longmore J., Tajti J., Uddman R., Edvinsson L. (2001). 5-HT(1B) and 5-HT(1D) receptors in the human trigeminal ganglion: Co-localization with calcitonin gene-related peptide, substance P and nitric oxide synthase. Brain Res..

[115] Thalakoti S., Patil V.V., Damodaram S., Vause C.V., Langford L.E., Freeman S.E., Durham P.L. (2007). Neuron-glia signaling in trigeminal ganglion: Implications for migraine pathology. Headache.

[116] Edvinsson J.C.A., Warfvinge K., Krause D.N., Blixt F.W., Sheykhzade M., Edvinsson L., Haanes K.A. (2019). C-fibers may modulate adjacent Aδ-fibers through axon-axon CGRP signaling at nodes of Ranvier in the trigeminal system. J. Headache Pain.

[117] Durham P.L., Russo A.F. (1999). Regulation of calcitonin gene-related peptide secretion by a serotonergic antimigraine drug. J. Neurosci. Off. J. Soc. Neurosci..

[118] Russell F.A., King R., Smillie S.J., Kodji X., Brain S.D. (2014). Calcitonin gene-related peptide: Physiology and pathophysiology. Physiol. Rev..

[119] Storer R.J., Akerman S., Goadsby P.J. (2004). Calcitonin gene-related peptide (CGRP) modulates nociceptive trigeminovascular transmission in the cat. Br. J. Pharmacol..

[120] Summ O., Charbit A.R., Andreou A.P., Goadsby P.J. (2010). Modulation of nocioceptive transmission with calcitonin gene-related peptide receptor antagonists in the thalamus. Brain J. Neurol..

[121] Pozo-Rosich P., Storer R.J., Charbit A.R., Goadsby P.J. (2015). Periaqueductal gray calcitonin gene-related peptide modulates trigeminovascular neurons. Cephalalgia.

[122] Recober A., Kuburas A., Zhang Z., Wemmie J.A., Anderson M.G., Russo A.F. (2009). Role of calcitonin gene-related peptide in light-aversive behavior: Implications for migraine. J. Neurosci. Off. J. Soc. Neurosci..

[123] Russo A.F., Kuburas A., Kaiser E.A., Raddant A.C., Recober A. (2009). A Potential Preclinical Migraine Model: CGRP-Sensitized Mice. Mol. Cell. Pharmacol..

[124] Recober A., Kaiser E.A., Kuburas A., Russo A.F. (2010). Induction of multiple photophobic behaviors in a transgenic mouse sensitized to CGRP. Neuropharmacology.

[125] Noseda R., Kainz V., Jakubowski M., Gooley J.J., Saper C.B., Digre K., Burstein R. (2010). A neural mechanism for exacerbation of headache by light. Nat. Neurosci..

[126] Zhang Z., Winborn C.S., Marquez de Prado B., Russo A.F. (2007). Sensitization of calcitonin gene-related peptide receptors by receptor activity-modifying protein-1 in the trigeminal ganglion. J. Neurosci. Off. J. Soc. Neurosci..

[127] Roon K.I., Olesen J., Diener H.C., Ellis P., Hettiarachchi J., Poole P.H., Christianssen I., Kleinermans D., Kok J.G., Ferrari M.D. (2000). No acute antimigraine efficacy of CP-122,288, a highly potent inhibitor of neurogenic inflammation: Results of two randomized, double-blind, placebo-controlled clinical trials. Ann. Neurol..

[128] May A., Goadsby P.J. (2001). Substance P receptor antagonists in the therapy of migraine. Expert. Opin. Investig. Drugs.

[129] Russo A.F. (2015). Calcitonin gene-related peptide (CGRP): A new target for migraine. Annu. Rev. Pharmacol. Toxicol..

[130] Ho T.W., Edvinsson L., Goadsby P.J. (2010). CGRP and its receptors provide new insights into migraine pathophysiology. Nat. Rev. Neurol..

[131] Tfelt-Hansen P., Le H. (2009). Calcitonin gene-related peptide in blood: Is it increased in the external jugular vein during migraine and cluster headache? A review. J. Headache Pain.

[132] Ashina M., Bendtsen L., Jensen R., Schifter S., Olesen J. (2000). Evidence for increased plasma levels of calcitonin gene-related peptide in migraine outside of attacks. Pain.

[133] Cernuda-Morollon E., Larrosa D., Ramon C., Vega J., Martinez-Camblor P., Pascual J. (2013). Interictal increase of CGRP levels in peripheral blood as a biomarker for chronic migraine. Neurology.

[134] Petersen K.A., Lassen L.H., Birk S., Lesko L., Olesen J. (2005). BIBN4096BS antagonizes human alpha-calcitonin gene related peptide-induced headache and extracerebral artery dilatation. Clin. Pharmacol. Ther..

[135] Christensen C.E., Younis S., Deen M., Khan S., Ghanizada H., Ashina M. (2018). Migraine induction with calcitonin gene-related peptide in patients from erenumab trials. J. Headache Pain.

[136] Guo S., Vollesen A.L., Olesen J., Ashina M. (2016). Premonitory and nonheadache symptoms induced by CGRP and PACAP38 in patients with migraine. Pain.

[137] Asghar M.S., Hansen A.E., Amin F.M., van der Geest R.J., Koning P., Larsson H.B., Olesen J., Ashina M. (2011). Evidence for a vascular factor in migraine. Ann. Neurol..

[138] Trasforini G., Margutti A., Portaluppi F., Menegatti M., Ambrosio M.R., Bagni B., Pansini R., Degli Uberti E.C. (1991). Circadian profile of plasma calcitonin gene-related peptide in healthy man. J. Clin. Endocrinol. Metab..

[139] Portaluppi F., Trasforini G., Margutti A., Vergnani L., Ambrosio M.R., Rossi R., Bagni B., Pansini R., degli Uberti E.C. (1992). Circadian rhythm of calcitonin gene-related peptide in uncomplicated essential hypertension. J. Hypertens..

[140] Fox A.W., Davis R.L. (1998). Migraine chronobiology. Headache.

[141] Ho T.W., Ferrari M.D., Dodick D.W., Galet V., Kost J., Fan X., Leibensperger H., Froman S., Assaid C., Lines C. (2008). Efficacy and tolerability of MK-0974 (telcagepant), a new oral antagonist of calcitonin gene-related peptide receptor, compared with zolmitriptan for acute migraine: A randomised, placebo-controlled, parallel-treatment trial. Lancet.

[142] Ho T.W., Connor K.M., Zhang Y., Pearlman E., Koppenhaver J., Fan X., Lines C., Edvinsson L., Goadsby P.J., Michelson D. (2014). Randomized controlled trial of the CGRP receptor antagonist telcagepant for migraine prevention. Neurology.

[143] Connor K.M., Aurora S.K., Loeys T., Ashina M., Jones C., Giezek H., Massaad R., Williams-Diaz A., Lines C., Ho T.W. (2011). Long-term tolerability of telcagepant for acute treatment of migraine in a randomized trial. Headache.

[144] Connor K.M., Shapiro R.E., Diener H.C., Lucas S., Kost J., Fan X., Fei K., Assaid C., Lines C., Ho T.W. (2009). Randomized, controlled trial of telcagepant for the acute treatment of migraine. Neurology.

[145] Hewitt D.J., Aurora S.K., Dodick D.W., Goadsby P.J., Ge Y.J., Bachman R., Taraborelli D., Fan X., Assaid C., Lines C. (2011). Randomized controlled trial of the CGRP receptor antagonist MK-3207 in the acute treatment of migraine. Cephalalgia.

[146] Olesen J., Diener H.C., Husstedt I.W., Goadsby P.J., Hall D., Meier U., Pollentier S., Lesko L.M. (2004). Calcitonin gene-related peptide receptor antagonist BIBN 4096 BS for the acute treatment of migraine. N. Engl. J. Med..

[147] Croop R., Lipton R.B., Kudrow D., Stock D.A., Kamen L., Conway C.M., Stock E.G., Coric V., Goadsby P.J. (2021). Oral rimegepant for preventive treatment of migraine: A phase 2/3, randomised, double-blind, placebo-controlled trial. Lancet.

[148] Hutchinson S., Schim J., Lipton R., Croop R., Jensen C., Thiry A., Stock E., Conway C., Lovegren M., Coric V. Oral rimegepant 75 mg is safe and well tolerated in adults with migraine and cardiovascular risk factors: Results of a multicenter, long-term, open-label safety study. Proceedings of the American Academy of Neurology 2021 Virtual Annual Meeting.

[149] Croop R., Goadsby P.J., Stock D.A., Conway C.M., Forshaw M., Stock E.G., Coric V., Lipton R.B. (2019). Efficacy, safety, and tolerability of rimegepant orally disintegrating tablet for the acute treatment of migraine: A randomised, phase 3, double-blind, placebo-controlled trial. Lancet.

[150] Lipton R.B., Coric V., Stock E.G., Stock D.A., Morris B.A., McCormack T.J. (2018). Rimegepant 75 mg, an oral calcitonin gene-related peptide antagonist, for the acute treatment of migraine: Two phase 3, double-blind, randomized, placebo-controlled trials. Cephalalgia.

[151] Hutchinson S., Silberstein S.D., Blumenfeld A.M., Lipton R.B., Lu K., Yu S.Y., Severt L. (2021). Safety and efficacy of ubrogepant in participants with major cardiovascular risk factors in two single-attack phase 3 randomized trials: ACHIEVE I and II. Cephalalgia.

[152] Hutchinson S., Dodick D.W., Treppendahl C., Bennett N.L., Yu S.Y., Guo H., Trugman J.M. (2021). Ubrogepant for the Acute Treatment of Migraine: Pooled Efficacy, Safety, and Tolerability from the ACHIEVE I and ACHIEVE II Phase 3 Randomized Trials. Neurol. Ther..

[153] Ailani J., Lipton R.B., Hutchinson S., Knievel K., Lu K., Butler M., Yu S.Y., Finnegan M., Severt L., Trugman J.M. (2020). Long-Term Safety Evaluation of Ubrogepant for the Acute Treatment of Migraine: Phase 3, Randomized, 52-Week Extension Trial. Headache.

[154] Dodick D.W., Lipton R.B., Ailani J., Halker Singh R.B., Shewale A.R., Zhao S., Trugman J.M., Yu S.Y., Viswanathan H.N. (2020). Ubrogepant, an Acute Treatment for Migraine, Improved Patient-Reported Functional Disability and Satisfaction in 2 Single-Attack Phase 3 Randomized Trials, ACHIEVE I and II. Headache.

[155] Lipton R.B., Dodick D.W., Ailani J., Lu K., Finnegan M., Szegedi A., Trugman J.M. (2019). Effect of Ubrogepant vs Placebo on Pain and the Most Bothersome Associated Symptom in the Acute Treatment of Migraine: The ACHIEVE II Randomized Clinical Trial. Jama.

[156] Dodick D.W., Lipton R.B., Ailani J., Lu K., Finnegan M., Trugman J.M., Szegedi A. (2019). Ubrogepant for the Treatment of Migraine. N. Engl. J. Med..

[157] Schwedt T.J., Lipton R.B., Ailani J., Silberstein S.D., Tassorelli C., Guo H., Lu K., Dabruzzo B., Miceli R., Severt L. (2022). Time course of efficacy of atogepant for the preventive treatment of migraine: Results from the randomized, double-blind ADVANCE trial. Cephalalgia.

[158] Boinpally R., Jakate A., Butler M., Periclou A. (2022). Atogepant and sumatriptan: No clinically relevant drug-drug interactions in a randomized, open-label, crossover trial. Pain Manag..

[159] Pozo-Rosich P., Ailani J., Ashina M., Goadsby P.J., Lipton R.B., Reuter U. (2023). Atogepant for the preventive treatment of chronic migraine: Results from the PROGRESS phase 3 trial. Neurology.

[160] Goadsby P.J., Dodick D.W., Ailani J., Trugman J.M., Finnegan M., Lu K., Szegedi A. (2020). Safety, tolerability, and efficacy of orally administered atogepant for the prevention of episodic migraine in adults: A double-blind, randomised phase 2b/3 trial. Lancet Neurol..

[161] Croop R., Madonia J., Stock D.A., Thiry A., Forshaw M., Murphy A., Coric V., Lipton R.B. (2022). Zavegepant nasal spray for the acute treatment of migraine: A Phase 2/3 double-blind, randomized, placebo-controlled, dose-ranging trial. Headache.

[162] Lipton R.B., Croop R., Stock D.A., Madonia J., Forshaw M., Lovegren M., Mosher L., Coric V., Goadsby P.J. (2023). Safety, tolerability, and efficacy of zavegepant 10 mg nasal spray for the acute treatment of migraine in the USA: A phase 3, double-blind, randomised, placebo-controlled multicentre trial. Lancet Neurol..

[163] Woodhead J.L., Siler S.Q., Howell B.A., Watkins P.B., Conway C. (2022). Comparing the Liver Safety Profiles of Four Next-Generation CGRP Receptor Antagonists to the Hepatotoxic CGRP Inhibitor Telcagepant Using Quantitative Systems Toxicology Modeling. Toxicol. Sci..

[164] Lipton R.B., Blumenfeld A., Jensen C.M., Croop R., Thiry A., L’Italien G., Morris B.A., Coric V., Goadsby P.J. (2023). Efficacy of rimegepant for the acute treatment of migraine based on triptan treatment experience: Pooled results from three phase 3 randomized clinical trials. Cephalalgia.

[165] Blumenfeld A.M., Goadsby P.J., Dodick D.W., Hutchinson S., Liu C., Finnegan M., Trugman J.M., Szegedi A. (2021). Efficacy of ubrogepant based on prior exposure and response to triptans: A post hoc analysis. Headache.

[166] Dodick D., Goadsby P.J., Schwedt T., Lipton R., Liu C., Lu K., Yu S.Y., Severt L., Finnegan M., Trugman J.M. (2023). Ubrogepant for the acute treatment of migraine when administered during the prodrome of migraine when administered during the prodrome (premonitory phase): Results from a phase 3, randomized, double-blind, placebo-controlled, crossover study. Headache.

[167] Goadsby P.J., Ailani J., Dodick D., Starling A., Liu C., Yu S.Y., Brand-Schieber E., Finnegan M., Trugman J.M. (2023). Efficacy of Ubrogepant for the Treatment of Migraine Symptoms During the Prodrome (Premonitory Phase): Results From the PRODROME Trial. Headache.

[168] Silberstein S.D., Dodick D.W., Bigal M.E., Yeung P.P., Goadsby P.J., Blankenbiller T., Grozinski-Wolff M., Yang R., Ma Y., Aycardi E. (2017). Fremanezumab for the Preventive Treatment of Chronic Migraine. N. Engl. J. Med..

[169] Sun H., Dodick D.W., Silberstein S., Goadsby P.J., Reuter U., Ashina M., Saper J., Cady R., Chon Y., Dietrich J. (2016). Safety and efficacy of AMG 334 for prevention of episodic migraine: A randomised, double-blind, placebo-controlled, phase 2 trial. Lancet. Neurol..

[170] Bigal M.E., Edvinsson L., Rapoport A.M., Lipton R.B., Spierings E.L., Diener H.C., Burstein R., Loupe P.S., Ma Y., Yang R. (2015). Safety, tolerability, and efficacy of TEV-48125 for preventive treatment of chronic migraine: A multicentre, randomised, double-blind, placebo-controlled, phase 2b study. Lancet Neurol..

[171] Bigal M.E., Walter S., Bronson M., Alibhoy A., Escandon R. (2014). Cardiovascular and hemodynamic parameters in women following prolonged CGRP inhibition using LBR-101, a monoclonal antibody against CGRP. Cephalalgia.

[172] Dodick D.W., Goadsby P.J., Silberstein S.D., Lipton R.B., Olesen J., Ashina M., Wilks K., Kudrow D., Kroll R., Kohrman B. (2014). Safety and efficacy of ALD403, an antibody to calcitonin gene-related peptide, for the prevention of frequent episodic migraine: A randomised, double-blind, placebo-controlled, exploratory phase 2 trial. Lancet Neurol..

[173] Skljarevski V., Oakes T.M., Zhang Q., Ferguson M.B., Martinez J., Camporeale A., Johnson K.W., Shan Q., Carter J., Schacht A. (2018). Effect of Different Doses of Galcanezumab vs Placebo for Episodic Migraine Prevention: A Randomized Clinical Trial. JAMA Neurol..

[174] Oakes T.M.M., Skljarevski V., Zhang Q., Kielbasa W., Hodsdon M.E., Detke H.C., Camporeale A., Saper J.R. (2018). Safety of galcanezumab in patients with episodic migraine: A randomized placebo-controlled dose-ranging Phase 2b study. Cephalalgia.

[175] Stauffer V.L., Dodick D.W., Zhang Q., Carter J.N., Ailani J., Conley R.R. (2018). Evaluation of Galcanezumab for the Prevention of Episodic Migraine: The EVOLVE-1 Randomized Clinical Trial. JAMA Neurol..

[176] Skljarevski V., Matharu M., Millen B.A., Ossipov M.H., Kim B.K., Yang J.Y. (2018). Efficacy and safety of galcanezumab for the prevention of episodic migraine: Results of the EVOLVE-2 Phase 3 randomized controlled clinical trial. Cephalalgia.

[177] Detke H.C., Goadsby P.J., Wang S., Friedman D.I., Selzler K.J., Aurora S.K. (2018). Galcanezumab in chronic migraine: The randomized, double-blind, placebo-controlled REGAIN study. Neurology.

[178] Goadsby P.J., Reuter U., Hallström Y., Broessner G., Bonner J.H., Zhang F., Sapra S., Picard H., Mikol D.D., Lenz R.A. (2017). A Controlled Trial of Erenumab for Episodic Migraine. N. Engl. J. Med..

[179] Ashina S., Melo-Carrillo A., Toluwanimi A., Bolo N., Szabo E., Borsook D., Burstein R. (2023). Galcanezumab effects on incidence of headache after occurrence of triggers, premonitory symptoms, and aura in responders, non-responders, super-responders, and super non-responders. J. Headache Pain.

[180] Edvinsson L. (2008). CGRP blockers in migraine therapy: Where do they act?. Br. J. Pharmacol..

[181] Amin F.M., Hougaard A., Cramer S.P., Christensen C.E., Wolfram F., Larsson H.B.W., Ashina M. (2017). Intact blood-brain barrier during spontaneous attacks of migraine without aura: A 3T DCE-MRI study. Eur. J. Neurol..

[182] Hougaard A., Amin F.M., Christensen C.E., Younis S., Wolfram F., Cramer S.P., Larsson H.B.W., Ashina M. (2017). Increased brainstem perfusion, but no blood-brain barrier disruption, during attacks of migraine with aura. Brain J. Neurol..

[183] Schankin C.J., Maniyar F.H., Seo Y., Kori S., Eller M., Chou D.E., Blecha J., Murphy S.T., Hawkins R.A., Sprenger T. (2016). Ictal lack of binding to brain parenchyma suggests integrity of the blood-brain barrier for 11C-dihydroergotamine during glyceryl trinitrate-induced migraine. Brain J. Neurol..

[184] Christensen S.L., Ernstsen C., Olesen J., Kristensen D.M. (2020). No central action of CGRP antagonising drugs in the GTN mouse model of migraine. Cephalalgia.

[185] Ziegeler C., Mehnert J., Asmussen K., May A. (2020). Central effects of erenumab in migraine patients: An event-related functional imaging study. Neurology.

[186] VanderPluym J., Dodick D.W., Lipton R.B., Ma Y., Loupe P.S., Bigal M.E. (2018). Fremanezumab for preventive treatment of migraine: Functional status on headache-free days. Neurology.

[187] Felgenhauer K. (1974). Protein size and cerebrospinal fluid composition. Klin. Wochenschr..

[188] Raddant A.C., Russo A.F. (2011). Calcitonin gene-related peptide in migraine: Intersection of peripheral inflammation and central modulation. Expert Rev. Mol. Med..

[189] Fila M., Sobczuk A., Pawlowska E., Blasiak J. (2022). Epigenetic Connection of the Calcitonin Gene-Related Peptide and Its Potential in Migraine. Int. J. Mol. Sci..

[190] Al-Hassany L., Boucherie D.M., Creeney H., van Drie R.W.A., Farham F., Favaretto S., Gollion C., Grangeon L., Lyons H., Marschollek K. (2023). Future targets for migraine treatment beyond CGRP. J Headache Pain..

[191] Tajti J., Uddman R., Edvinsson L. (2001). Neuropeptide localization in the “migraine generator” region of the human brainstem. Cephalalgia.

[192] Uddman R., Tajti J., Hou M., Sundler F., Edvinsson L. (2002). Neuropeptide expression in the human trigeminal nucleus caudalis and in the cervical spinal cord C1 and C2. Cephalalgia.

[193] Laburthe M., Couvineau A. (2002). Molecular pharmacology and structure of VPAC Receptors for VIP and PACAP. Regul. Pept..

[194] Falktoft B., Georg B., Fahrenkrug J. (2009). Signaling pathways in PACAP regulation of VIP gene expression in human neuroblastoma cells. Neuropeptides.

[195] Georg B., Fahrenkrug J. (2000). Pituitary adelylate cyclase-activating peptide is an activator of vasoactive intestinal polypeptide gene transcription in human neuroblastoma cells. Brain Res. Mol. Brain Res..

[196] Guo S., Vollesen A.L., Hansen R.D., Esserlind A.L., Amin F.M., Christensen A.F., Olesen J., Ashina M. (2017). Part I: Pituitary adenylate cyclase-activating polypeptide-38 induced migraine-like attacks in patients with and without familial aggregation of migraine. Cephalalgia.

[197] Banks W.A., Kastin A.J., Komaki G., Arimura A. (1993). Passage of pituitary adenylate cyclase activating polypeptide1-27 and pituitary adenylate cyclase activating polypeptide1-38 across the blood-brain barrier. J. Pharmacol. Exp. Ther..

[198] Onderwater G.L.J., Dool J., Ferrari M.D., Terwindt G.M. (2020). Premonitory symptoms in glyceryl trinitrate triggered migraine attacks: A case-control study. Pain.

[199] Vaudry D., Falluel-Morel A., Bourgault S., Basille M., Burel D., Wurtz O., Fournier A., Chow B.K., Hashimoto H., Galas L. (2009). Pituitary adenylate cyclase-activating polypeptide and its receptors: 20 years after the discovery. Pharmacol. Rev..

[200] Schulte L.H., Mehnert J., May A. (2020). Longitudinal Neuroimaging over 30 Days: Temporal Characteristics of Migraine. Ann. Neurol..

[201] Kuburas A., Mason B.N., Hing B., Wattiez A.S., Reis A.S., Sowers L.P., Moldovan Loomis C., Garcia-Martinez L.F., Russo A.F. (2021). PACAP Induces Light Aversion in Mice by an Inheritable Mechanism Independent of CGRP. J. Neurosci. Off. J. Soc. Neurosci..

[202] De Logu F., Landini L., Janal M.N., Li Puma S., De Cesaris F., Geppetti P., Nassini R. (2019). Migraine-provoking substances evoke periorbital allodynia in mice. J. Headache Pain.

[203] Ernstsen C., Christensen S.L., Rasmussen R.H., Nielsen B.S., Jansen-Olesen I., Olesen J., Kristensen D.M. (2022). The PACAP pathway is independent of CGRP in mouse models of migraine: Possible new drug target?. Brain J. Neurol..

[204] Emery A.C., Eiden L.E. (2012). Signaling through the neuropeptide GPCR PAC₁ induces neuritogenesis via a single linear cAMP- and ERK-dependent pathway using a novel cAMP sensor. FASEB J..

[205] Goadsby P.J., Zagami A.S. (1991). Stimulation of the superior sagittal sinus increases metabolic activity and blood flow in certain regions of the brainstem and upper cervical spinal cord of the cat. Brain J. Neurol..

[206] Goadsby P.J., Edvinsson L. (1994). Human in vivo evidence for trigeminovascular activation in cluster headache. Neuropeptide changes and effects of acute attacks therapies. Brain J. Neurol..

[207] Wienholtz N.K.F., Christensen C.E., Zhang D.G., Coskun H., Ghanizada H., Al-Karagholi M.A., Hannibal J., Egeberg A., Thyssen J.P., Ashina M. (2021). Early treatment with sumatriptan prevents PACAP38-induced migraine: A randomised clinical trial. Cephalalgia.

[208] Amin F.M., Asghar M.S., Guo S., Hougaard A., Hansen A.E., Schytz H.W., van der Geest R.J., de Koning P.J., Larsson H.B., Olesen J. (2012). Headache and prolonged dilatation of the middle meningeal artery by PACAP38 in healthy volunteers. Cephalalgia.

[209] Amin F.M., Hougaard A., Magon S., Asghar M.S., Ahmad N.N., Rostrup E., Sprenger T., Ashina M. (2016). Change in brain network connectivity during PACAP38-induced migraine attacks: A resting-state functional MRI study. Neurology.

[210] Ghanizada H., Al-Karagholi M.A., Arngrim N., Olesen J., Ashina M. (2020). PACAP27 induces migraine-like attacks in migraine patients. Cephalalgia.

[211] Rasmussen N.B., Deligianni C., Christensen C.E., Karlsson W.K., Al-Khazali H.M., Van de Casteele T., Granhall C., Amin F.M., Ashina M. (2023). The effect of Lu AG09222 on PACAP38- and VIP-induced vasodilation, heart rate increase, and headache in healthy subjects: An interventional, randomized, double-blind, parallel-group, placebo-controlled study. J. Headache Pain.

[212] Edvinsson L. (2013). Role of VIP/PACAP in primary headaches. Cephalalgia.

[213] Goadsby P.J., MacDonald G.J. (1985). Extracranial vasodilation mediated by vasoactive intestinal polypeptide (VIP). Brain Res..

[214] Jansen-Olesen I., Baun M., Amrutkar D.V., Ramachandran R., Christophersen D.V., Olesen J. (2014). PACAP-38 but not VIP induces release of CGRP from trigeminal nucleus caudalis via a receptor distinct from the PAC1 receptor. Neuropeptides.

[215] Mason B.N., Wattiez A.S., Balcziak L.K., Kuburas A., Kutschke W.J., Russo A.F. (2020). Vascular actions of peripheral CGRP in migraine-like photophobia in mice. Cephalalgia.

[216] Tore F., Korkmaz O.T., Dogrukol-Ak D., Tunçel N. (2010). The effects of vasoactive intestinal peptide on dura mater nitric oxide levels and vessel-contraction responses in sympathectomized rats. J. Mol. Neurosci..

[217] Kilinc E., Firat T., Tore F., Kiyan A., Kukner A., Tunçel N. (2015). Vasoactive Intestinal peptide modulates c-Fos activity in the trigeminal nucleus and dura mater mast cells in sympathectomized rats. J. Neurosci. Res..

[218] Cernuda-Morollón E., Martínez-Camblor P., Alvarez R., Larrosa D., Ramón C., Pascual J. (2015). Increased VIP levels in peripheral blood outside migraine attacks as a potential biomarker of cranial parasympathetic activation in chronic migraine. Cephalalgia.

[219] Bellamy J.L., Cady R.K., Durham P.L. (2006). Salivary levels of CGRP and VIP in rhinosinusitis and migraine patients. Headache.

[220] Sarchielli P., Pini L.A., Zanchin G., Alberti A., Maggioni F., Rossi C., Floridi A., Calabresi P. (2006). Clinical-biochemical correlates of migraine attacks in rizatriptan responders and non-responders. Cephalalgia.

[221] Cernuda-Morollón E., Martínez-Camblor P., Ramón C., Larrosa D., Serrano-Pertierra E., Pascual J. (2014). CGRP and VIP levels as predictors of efficacy of Onabotulinumtoxin type A in chronic migraine. Headache.

[222] Hansen J.M., Sitarz J., Birk S., Rahmann A.M., Oturai P.S., Fahrenkrug J., Olesen J., Ashina M. (2006). Vasoactive intestinal polypeptide evokes only a minimal headache in healthy volunteers. Cephalalgia.

[223] Riesco N., Cernuda-Morollón E., Martínez-Camblor P., Pérez-Alvarez A.I., Verano L., García-Cabo C., Serrano-Pertierra E., Pascual J. (2017). Relationship between serum levels of VIP, but not of CGRP, and cranial autonomic parasympathetic symptoms: A study in chronic migraine patients. Cephalalgia.

[224] Pellesi L., Al-Karagholi M.A., Chaudhry B.A., Lopez C.L., Snellman J., Hannibal J., Amin F.M., Ashina M. (2020). Two-hour infusion of vasoactive intestinal polypeptide induces delayed headache and extracranial vasodilation in healthy volunteers. Cephalalgia.

[225] Karsan N., Nagaraj K., Goadsby P.J. (2022). Cranial autonomic symptoms: Prevalence, phenotype and laterality in migraine and two potentially new symptoms. J. Headache Pain.

[226] Mason B.N., Kaiser E.A., Kuburas A., Loomis M.M., Latham J.A., Garcia-Martinez L.F., Russo A.F. (2017). Induction of Migraine-Like Photophobic Behavior in Mice by Both Peripheral and Central CGRP Mechanisms. J. Neurosci. Off. J. Soc. Neurosci..

[227] van den Pol A.N. (2012). Neuropeptide transmission in brain circuits. Neuron.

[228] Edvinsson L., Copeland J.R., Emson P.C., McCulloch J., Uddman R. (1987). Nerve Fibers Containing Neuropeptide Y in the Cerebrovascular Bed: Immunocytochemistry, Radioimmunoassay, and Vasomotor Effects. J. Cereb. Blood Flow Metab..

[229] Parker R.M., Herzog H. (1999). Regional distribution of Y-receptor subtype mRNAs in rat brain. Eur. J. Neurosci..

[230] Stanley B.G., Kyrkouli S.E., Lampert S., Leibowitz S.F. (1986). Neuropeptide Y chronically injected into the hypothalamus: A powerful neurochemical inducer of hyperphagia and obesity. Peptides.

[231] Yamanaka A., Kunii K., Nambu T., Tsujino N., Sakai A., Matsuzaki I., Miwa Y., Goto K., Sakurai T. (2000). Orexin-induced food intake involves neuropeptide Y pathway. Brain Res..

[232] Wang J.H., Wang F., Yang M.J., Yu D.F., Wu W.N., Liu J., Ma L.Q., Cai F., Chen J.G. (2008). Leptin regulated calcium channels of neuropeptide Y and proopiomelanocortin neurons by activation of different signal pathways. Neuroscience.

[233] Sim L.J., Joseph S.A. (1991). Arcuate nucleus projections to brainstem regions which modulate nociception. J. Chem. Neuroanat..

[234] Kuphal K.E., Solway B., Pedrazzini T., Taylor B.K. (2008). Y1 receptor knockout increases nociception and prevents the anti-allodynic actions of NPY. Nutrition.

[235] Holland P., Goadsby P.J. (2007). The hypothalamic orexinergic system: Pain and primary headaches. Headache.

[236] Holland P.R., Akerman S., Goadsby P.J. (2006). Modulation of nociceptive dural input to the trigeminal nucleus caudalis via activation of the orexin 1 receptor in the rat. Eur. J. Neurosci..

[237] Holland P.R., Akerman S., Goadsby P.J. (2005). Orexin 1 receptor activation attenuates neurogenic dural vasodilation in an animal model of trigeminovascular nociception. J. Pharmacol. Exp. Ther..

[238] Bartsch T., Levy M.J., Knight Y.E., Goadsby P.J. (2004). Differential modulation of nociceptive dural input to [hypocretin] orexin A and B receptor activation in the posterior hypothalamic area. Pain.

[239] Hoffmann J., Supronsinchai W., Akerman S., Andreou A.P., Winrow C.J., Renger J., Hargreaves R., Goadsby P.J. (2015). Evidence for orexinergic mechanisms in migraine. Neurobiol. Dis..

[240] Martins-Oliveira M., Akerman S., Tavares I., Goadsby P.J. (2016). Neuropeptide Y inhibits the trigeminovascular pathway through NPY Y1 receptor: Implications for migraine. Pain.

[241] Guo Y., Cheng Y., An J., Qi Y., Luo G. (2021). Neuropeptide changes in an improved migraine model with repeat stimulations. Transl. Neurosci..

[242] Yang C., Gong Z., Zhang X., Miao S., Li B., Xie W., Wang T., Han X., Wang L., Dong Z. (2023). Neuropeptide Y in the medial habenula alleviates migraine-like behaviors through the Y1 receptor. J. Headache Pain.

[243] Kushi A., Sasai H., Koizumi H., Takeda N., Yokoyama M., Nakamura M. (1998). Obesity and mild hyperinsulinemia found in neuropeptide Y-Y1 receptor-deficient mice. Proc. Natl. Acad. Sci. USA.

[244] Martins-Oliveira M., Akerman S., Holland P.R., Hoffmann J.R., Tavares I., Goadsby P.J. (2017). Neuroendocrine signaling modulates specific neural networks relevant to migraine. Neurobiol. Dis..

[245] Szentirmai E., Krueger J.M. (2006). Central administration of neuropeptide Y induces wakefulness in rats. Am. J. Physiol. Regul. Integr. Comp. Physiol..

[246] Tóth A., Hajnik T., Záborszky L., Détári L. (2007). Effect of basal forebrain neuropeptide Y administration on sleep and spontaneous behavior in freely moving rats. Brain Res. Bull..

[247] Heilig M. (2004). The NPY system in stress, anxiety and depression. Neuropeptides.

[248] Gallai V., Sarchielli P., Trequattrini A., Paciaroni M., Usai F., Palumbo R. (1994). Neuropeptide Y in juvenile migraine and tension-type headache. Headache.

[249] Caproni S., Corbelli I., Pini L.A., Cupini M.L., Calabresi P., Sarchielli P. (2011). Migraine preventive drug-induced weight gain may be mediated by effects on hypothalamic peptides: The results of a pilot study. Cephalalgia.

[250] Frederiksen S.D., Bekker-Nielsen Dunbar M., Snoer A.H., Deen M., Edvinsson L. (2020). Serotonin and Neuropeptides in Blood From Episodic and Chronic Migraine and Cluster Headache Patients in Case-Control and Case-Crossover Settings: A Systematic Review and Meta-Analysis. Headache.

[251] Vécsei L., Widerlöv E., Ekman R., Kovács K., Jelencsik I., Bozsik G., Kapócs G. (1992). Suboccipital cerebrospinal fluid and plasma concentrations of somatostatin, neuropeptide Y and beta-endorphin in patients with common migraine. Neuropeptides.

[252] Valenzuela R.F., Donoso M.V., Mellado P.A., Huidobro-Toro J.P. (2000). Migraine, but not subarachnoid hemorrhage, is associated with differentially increased NPY-like immunoreactivity in the CSF. J. Neurol. Sci..

[253] Chabi A., Zhang Y., Jackson S., Cady R., Lines C., Herring W.J., Connor K.M., Michelson D. (2015). Randomized controlled trial of the orexin receptor antagonist filorexant for migraine prophylaxis. Cephalalgia.

[254] Karsan N., Bose P., Newman J., Goadsby P.J. (2021). Are some patient-perceived migraine triggers simply early manifestations of the attack?. J. Neurol..

[255] Winter A.C., Berger K., Buring J.E., Kurth T. (2009). Body mass index, migraine, migraine frequency and migraine features in women. Cephalalgia.

[256] Martins-Oliveira M., Tavares I., Goadsby P.J. (2021). Was it something I ate? Understanding the bidirectional interaction of migraine and appetite neural circuits. Brain Res..

[257] Siva Z.O., Uluduz D., Keskin F.E., Erenler F., Balcı H., Uygunoğlu U., Saip S., Göksan B., Siva A. (2018). Determinants of glucose metabolism and the role of NPY in the progression of insulin resistance in chronic migraine. Cephalalgia.

[258] Held K., Antonijevic I., Murck H., Kuenzel H., Steiger A. (2006). Neuropeptide Y (NPY) shortens sleep latency but does not suppress ACTH and cortisol in depressed patients and normal controls. Psychoneuroendocrinology.

[259] Karsan N., Goadsby P.J. (2021). Migraine Is More Than Just Headache: Is the Link to Chronic Fatigue and Mood Disorders Simply Due to Shared Biological Systems?. Front. Hum. Neurosci..

[260] Meye F.J., Adan R.A. (2014). Feelings about food: The ventral tegmental area in food reward and emotional eating. Trends Pharmacol. Sci..

[261] Martins-Oliveira M., Akerman S., Holland P.R., Tavares I., Goadsby P.J. (2022). Pharmacological modulation of ventral tegmental area neurons elicits changes in trigeminovascular sensory processing and is accompanied by glycemic changes: Implications for migraine. Cephalalgia.

[262] Bernecker C., Pailer S., Kieslinger P., Horejsi R., Möller R., Lechner A., Wallner-Blazek M., Weiss S., Fazekas F., Truschnig-Wilders M. (2010). GLP-2 and leptin are associated with hyperinsulinemia in non-obese female migraineurs. Cephalalgia.

[263] Goldstein D.J., Offen W.W., Klein E.G., Phebus L.A., Hipskind P., Johnson K.W., Ryan R.E. (2001). Lanepitant, an NK-1 antagonist, in migraine prevention. Cephalalgia.

[264] Goldstein D.J., Wang O., Saper J.R., Stoltz R., Silberstein S.D., Mathew N.T. (1997). Ineffectiveness of neurokinin-1 antagonist in acute migraine: A crossover study. Cephalalgia.

[265] Tajti J., Szok D., Majlath Z., Tuka B., Csati A., Vecsei L. (2015). Migraine and neuropeptides. Neuropeptides.

[266] Snoer A., Lund N., Beske R., Hagedorn A., Jensen R.H., Barloese M. (2018). Cluster headache beyond the pain phase: A prospective study of 500 attacks. Neurology.

[267] Snoer A., Lund N., Beske R., Jensen R., Barloese M. (2018). Pre-attack signs and symptoms in cluster headache: Characteristics and time profile. Cephalalgia.

